# Lipases and carboxylesterases affect moth sex pheromone compounds involved in interspecific mate recognition

**DOI:** 10.1038/s41467-023-43100-w

**Published:** 2023-11-18

**Authors:** Arthur de Fouchier, Elise Fruitet, Rik Lievers, Peter Kuperus, Jennifer Emerson, Fred Gould, David G. Heckel, Astrid T. Groot

**Affiliations:** 1 Institute for Biodiversity and Ecosystem Dynamics, University of Amsterdam, Science Park 904, Amsterdam, the Netherlands; 2 Department of Entomology, Max Planck Institute for Chemical Ecology, Hans-Knöll-Strasse 8, Jena, Germany; 3 Department of Entomology and Plant Pathology, North Carolina State University, Raleigh, NC 27695 USA; 4 Present Address: Institute of Ecology and Environmental Sciences of Paris, Sorbonne Université, INRAE, CNRS, IRD, UPEC, Université de Paris, Paris, France

**Keywords:** Chemical ecology, Molecular evolution

## Abstract

Moth sex pheromones are a classical model for studying sexual selection. Females typically produce a species-specific pheromone blend that attracts males. Revealing the enzymes involved in the interspecific variation in blend composition is key for understanding the evolution of these sexual communication systems. The nature of the enzymes involved in the variation of acetate esters, which are prominent compounds in moth pheromone blends, remains unclear. We identify enzymes involved in acetate degradation using two closely related moth species: *Heliothis* (*Chloridea*) *subflexa* and *H*. (*C.*) *virescens*, which have different quantities of acetate esters in their sex pheromone. Through comparative transcriptomic analyses and CRISPR/Cas9 knockouts, we show that two lipases and two esterases from *H. virescens* reduce the levels of pheromone acetate esters when expressed in *H. subflexa* females. Together, our results show that lipases and carboxylesterases are involved in tuning Lepidoptera pheromones composition.

## Abstract (web summary)

Moth sex pheromones, pivotal for mate attraction, are a classic model for sexual selection. Through introgression, transcriptomics and knocking out genes, this study identifies lipases and esterases that affect pheromone blend composition, offering insights into moth pheromone evolution.

## Introduction

One of the most fascinating questions in evolutionary biology is to understand how new species arise. Sexual signals, through which individuals recognize suitable mating partners, can have a critical role in prezygotic isolation of species. As such, sexual signals offer a seductive model to uncover evolutionary patterns that lead to speciation^1^. Identifying genes underlying the production and detection of sexual signals opens a door to the analysis of their evolution across species.

Pheromones are semiochemicals released by a sender that modify the behavior of a conspecific receiver. Sex pheromones are signals used in the context of mate finding or courtship^2^. In most nocturnal moths, females typically produce a species-specific blend of a small number of so-called type I sex pheromone^3^ components to which conspecific males are attracted^4^. The species-specificity of moth sex pheromone blends is mainly determined by the ratio of alcohols, aldehydes, and acetate esters (hereafter also referred to as “acetates”) with carbon backbones of various lengths and degrees of desaturation^4,5^. Since the sex pheromones of more than 2000 moth species have been identified^6^, moths have become exemplary models to understand the evolution of sexual communication. In sympatric, ecologically similar species with similar pheromone blends, females may not only release attractive components, but also repellent pheromone components that prevent attraction of heterospecific males^7–11^. Such is the case with *Heliothis (Chloridea) subflexa* females, where males of the closely related *H. (C.) virescens* are repelled by the acetate esters in the *H. subflexa* pheromone^7,12,13^.

Despite 25 years of research in which techniques of molecular biology have been applied to pheromone biosynthetic enzymes^14,15^, the genes involved in regulating the proportion of acetates in the blends have not been identified yet^16^. This is surprising, because a phylogenetic survey covering 1572 moth species in 619 genera and 49 families, shows that five different acetates are the five most commonly occurring components of moth pheromones^17^. Biochemical studies have shown that microsomal fractions of female pheromone glands of the spruce budworm *Choristoneura fumiferana*^18,19^ and two other tortricids as well as the crambid *Ostrinia nubilalis*^20^ can conjugate a wide variety of fatty alcohols to acetate esters provided by acetyl-CoA through acetyltransferase activity.

Previous attempts to link candidate genes to acetyltransferase activity include a study in which expression differences of candidate acetyltransferase genes failed to correlate with the presence/absence of acetates in the two closely related and sympatrically occurring species *H. virescens* and *H. subflexa*^21^. Also notable is a study in which 34 candidate genes from *Agrotis segetum* failed to yield acetate esters in a yeast expression system^16^. Misleadingly, each of these 34 genes is now annotated as “fatty alcohol acetyltransferase” in GenBank (Accession Numbers KJ579206–KJ579239). This precedent has led to paralogs in other species being named as acetyltransferases, despite the absence of any biochemical evidence (e.g., *Helicoverpa armigera* (Accession Numbers MF706167–MF706196) and *H. assulta* (Accession Numbers MF687638–MF687667^22^, *Antheraea pernyi* (ACT1-ACT22)^23^).

*Heliothis virescens* and *H. subflexa* are two closely related species occurring in sympatry. These noctuid moths have recently been reclassified from the paraphyletic genus *Heliothis* to the monophyletic genus *Chloridea*^24^ but we retain the older name here for continuity with the extensive literature on pheromones in these species. The most striking pheromone difference between these two species is the presence of (Z)-11-hexadecenyl acetate (Z11-16:OAc) and two other acetate esters (Z7-16:OAc and Z9-16:OAc) in the *H. subflexa* female pheromone, which to our knowledge have never been found in the *H. virescens* female blend^6^. Previous behavioral experiments have shown that this difference plays a critical role in the reproductive isolation of these two species, because the presence of these acetates in the pheromone blend inhibits the attraction of *H. virescens* males while increasing *H.subflexa* male attraction^7,25,26^. Since these two species can be hybridized in captivity, they are an excellent model to identify the genes underlying acetate variation.

**Fig. 1:**
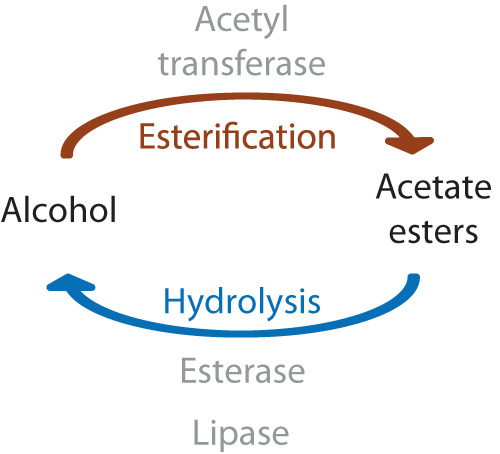
Balance between alcohol and acetate esters by enzymatic reactions. Schematic of the balance between the esterification of alcohol by acetyltransferases and the reverse reaction of hydrolysis of acetate esters into alcohol by enzymes with hydrolytic activity (esterase or lipase).

The quantity of acetates released by the female pheromone gland^27^ is most likely a balance between biosynthesis by conjugation of the acetyl moeity to a fatty alcohol, and degradation by hydrolysis of the ester back to the alcohol and acetic acid (Fig. 1). Our goal is to understand both processes by comparative and genetic studies of *H. subflexa* and *H. virescens*, which is complicated by the fact that, even though acetates have to our knowledge never been detected in the *H. virescens* female pheromone gland^6^, *H. virescens* males produce acetates in the hairpencil glands^28^ and both sexes contain acetates on their legs^29^. Both species therefore must possess one or more genes encoding acetyltransferase activity, making genomic comparisons inconclusive. Therefore, we have begun our investigations by searching for the genes responsible for hydrolysis, which determines the final quantity of acetates released from the originally synthesized amount.

In previous work, when backcrossing *H. virescens* to *H. subflexa*, two quantitative trait loci (QTL) were found that significantly contribute to the acetate reduction^30^. We consider these QTLs as candidates for genes whose products hydrolyze the acetates synthesized in the female pheromone gland. One of these *H. virescens* chromosomes was introgressed into the genetic background of *H. subflexa* by repeated backcrossing and screening using AFLP markers^31^. This produced the so-called DD23 population with individuals carrying one or no copies of *H. virescens* Chromosome 20 (HvirChr20) and one or two copies of the corresponding chromosome from *H. subflexa* (HsubChr20). All of the 30 other chromosomes had both copies from *H. subflexa*. During the initial backcrossing the entire HvirChr20 was introgressed into the *H. subflexa* genomic background by using backcross females, where no crossing-over takes place^31,32^. After 9 generations, backcross males were crossed to *H. subflexa* females, which resulted in just one end of HvirChr20 segregating in DD23 (Supplementary Fig. S1). This partial HvirChr20 introgression significantly reduced acetate levels in the female sex pheromone.

Here, we sequence cDNA from the pheromone glands of females homozygous for the HvirChr20 introgression (VV) and females homozygous for the wild-type HsubChr20 (SS) in the segregating DD23 population. Using a de novo assembled transcriptome, we find gene expression differences between transcripts from *H. virescens* and *H. subflexa* alleles of the introgressed region, and identify two lipase genes and two esterase genes from *H. virescens* as candidates contributing to acetate hydrolysis. We inactivate these *H. virescens* genes using CRISPR/Cas9 gene editing in females heterozygous for the HvirChr20 intogression (VS) and observe an increase in acetates. Homology modeling of the enzymes and docking studies with the three acetate esters produced by *H. subflexa* yields binding models consistent with hydrolytic activity. Overall, we find that these lipases and esterases significantly affect the final amounts of acetate esters in the females’ sex pheromone, which in the *H. subflexa*/*virescens* system, play a key role in reproductive isolation^7^.

## Results

### Dominant effect of *H. virescens* QTL for reduced acetate levels

**Fig. 2:**
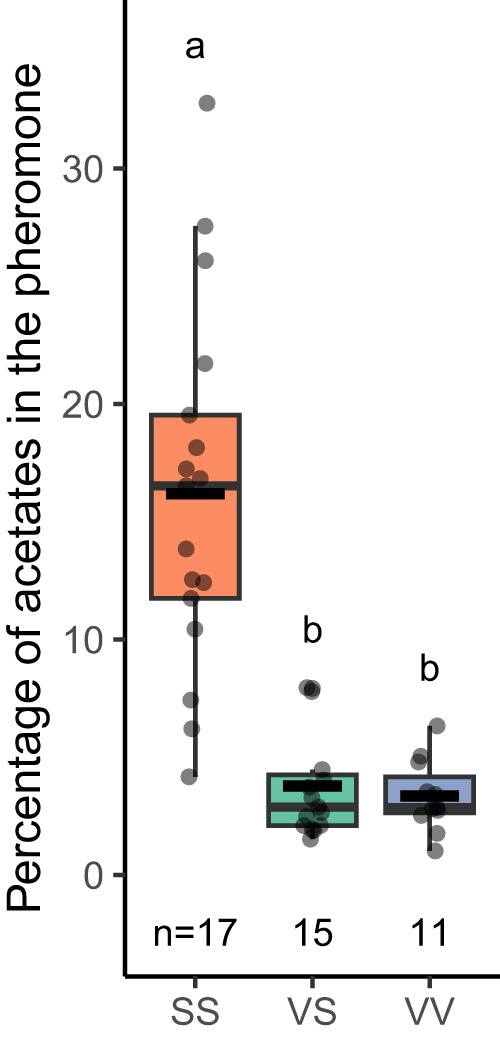
Acetate levels in introgressed lines. Boxplots of the acetate levels in the different genotypes of females from the DD23 line. VV females (purple) carry two copies of *H. virescens* QTL-Chr20, VS females (green) carry only one copy and SS females (orange) don’t have *H. virescens* introgression. Boxplots centers represent the median with lower and upper bounds representing the first and third quartiles, respectively. Lower and upper whiskers of the plots display values that are below the first quartile but not exceeding 1.5 times the interquartile range, and values above the third quartile but not exceeding 1.5 times the interquartile range. The incomplete horizontal black line represents the mean. All data points can be seen as partly transparent black dots. Letters represent group of statistical similarity based on Welch one-way ANOVA followed by Games-Howell post-hoc test (*p* = 0.000013 for SS-VS, 0.00000908 for SS-VV and 0.834 for VS-VV). Source data are provided as a Source data file.

Through continuous backcrossing one of the two major QTLs for acetate levels^30,31,33^, we isolated this QTL (2.4 cM at one end of Chromosome 20) into an otherwise complete *H. subflexa* genomic background. We refer to this introgressed line as DD23. VV (homozygous) and VS (heterozygous) DD23 females showed significantly lower acetate levels than SS (wild type) *H. subflexa* (Tukey post-hoc test, *p* < 0.001) (Fig. 2). Also, the introgression of Chr20-QTL from *H. virescens* into *H. subflexa* has a dominant effect, as VS females had similarly low levels of acetates as VV (Tukey post-hoc test, *p* = 0.585).

### Assembly of the DD23 pheromone gland reference transcriptome

We first generated a reference transcriptome through RNAseq reads assembly of RNA extracted from VV and SS glands. After filtering this transcriptome contained 18343 contigs, with an N50 of 2603 (Supplementary Table S1). To assess the transcriptome quality, we used the tool BUSCO and found 1480 (89.26%) of the 1658 BUSCO genes (Supplementary Table S1). 1247 Of these genes were found in a single complete copy and 233 were found in duplicated complete copy. We also used the Transrate tool that use alignment of the reads on the contigs to assess the quality of the assembly^34^. The overall assembly score was 0.317, while the unfiltered transcriptome assembly score was 0.247 and the mean optimized assembly score for arthropod transcriptomes tested by Transrate is 0.256^34^. The number of segmented contigs was 3560 in our reference transcriptome, with an uneven read coverage along their sequence, versus 14154 for the unfiltered transcriptome. These quality metrics led us to be confident about the quality of the DD23 pheromone gland transcriptome, which we used to perform a differential expression analysis.

### Identification in the DD23 pheromone gland reference transcriptome of candidate lipases and esterases putatively involved in the difference in acetate levels in the pheromone

To determine which genes were differentially expressed in the sex pheromone glands of the different types of females, we analyzed the transcriptomes of these glands after building a reference transcriptome (see Supplementary Results). We considered differentially expressed contigs when we found a significant log2 fold change in expression with an absolute value of 2 or more. This first filtering revealed 89 differentially expressed contigs, none of which were annotated by Blast2Go with putative functions in pheromone biosynthesis (see Supplementary Table S2). However, among these differentially expressed contigs, five were annotated by Blast2Go as putative lipases (38088_c0_seq4, 38088_c0_seq5, 38579_c0_seq3, 39873_c1_seq2 and 39873_c1_seq3) and one as putative esterase (comp39435_c1_seq17) (Supplementary Table S2). As the GO terms predicted that the genes represented by these contigs are putatively involved in acetate degradation, we considered these contigs as candidate transcripts for explaining the phenotypic difference in acetate levels between VV and SS.

### In vivo expression of candidate lipases and esterases

As a first step in verifying in silico observations, we confirmed the sequence of candidate transcripts by Sanger sequencing. Consistent with its low expression levels in the RNAseq experiment, comp38088_c0_seq5 could not be amplified from *H. subflexa* pheromone gland cDNA. Similarly, comp38579_c0_seq3 could not be amplified from *H. virescens* pheromone gland cDNA. The sequences resulting from Sanger sequencing were named: *LipX* (38088_c0_seq4; OK556469, OK55646970), *LipZ* (38088_c0_seq5; OK556475), *Est1* (comp39435_c1_seq17; OK556471, OK556472), *Lip39873* (39873_c1_seq2; OK556473, OK556474) and *Lip38579* (38579_c0_seq3; OK556476).

**Fig. 3:**
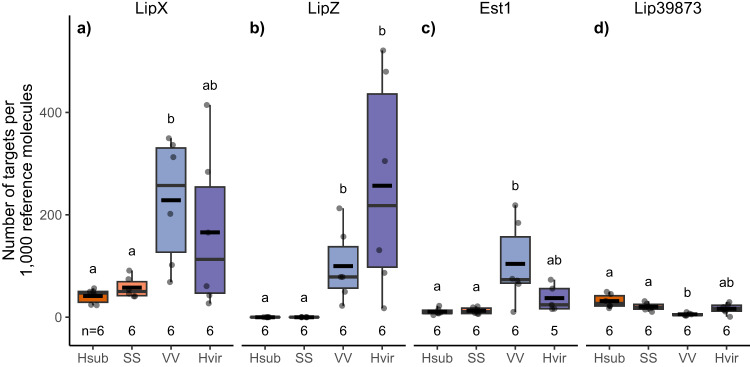
Expression levels of candidate genes in *H. subflexa*, *H. virescens*, and DD23 female pheromone glands by RT-qPCR. Boxplots of the expression of the transcripts of **a** *LipX*, **b** *LipZ*, **c** *Est1*, and **d** *Lip39873* in target transcripts per 1000 reference molecules measured by RT-qPCR in pheromone gland cDNA from *H. subflexa* (orange), *H. virescens* (purple), DD23 SS (light orange), and VV (light purple) females. Boxplots are built similarly as in Fig. 2. Letters represent groups of statistical similarity based on Welch one-way ANOVA followed by Tukey or Games-Howell post-hoc tests for *LipZ* and *Est1* or *LipX* and *Lip39873* respectively (exact *p*-values in Supplementary Table S3). Source data are provided as a Source data file.

We then verified the differential expression levels in the RNAseq experiment with quantitative real-time PCR experiments on pheromone gland cDNA, except for *Lip38579* for which we could not find primers with satisfactory efficiency. We observed higher expression levels of *LipX* and *Est1* in *H. virescens* and VV individuals compared to *H. subflexa* and SS females, and this difference was significant in comparing VV with *H. subflexa* and SS individuals (Games-Howell post-hoc test, *p* < 0.05, Supplementary Table S3; Fig. 3a, c). For *LipZ*, the expression level was significantly higher in the pheromone glands of VV and *H. virescens* females compared to SS and *H. subflexa* females (Tukey post-hoc test, *p* < 0.05, Supplementary Table S3; Fig. 3b). For *Lip39873*, we observed overall very low and similar expression levels in *H. subflexa*, *H. virescens* and SS female pheromone glands (Fig. 3d) and therefore did not consider this gene as a candidate for further experiments.

Based on our reference DD23 pheromone gland transcriptome, we observed a number of contigs that have the same best hit in nr (NCBI) and opposite over-expression patterns in the differential expression analysis. For the transcript we re-sequenced, we observed that our reference transcriptome included pairs of contigs matching either mostly the *H. subflexa* or the *H. virescens* alleles of the genes. The sequence divergence between homologous alleles give rise to mapping the reads to the contigs in a species-specific way. When we blasted the contigs considered as differentially expressed in Supplementary Table S2 against the *Bombyx mori* genome, we found that most their *Bombyx* best hits were distributed along one end of Chromosome 20 (Supplementary Table S4). Many of these Chromosome 20-mapping contigs occurred in pairs, one representing the *virescens* allele and the other representing the *subflexa* allele. Consequently, any gene with enough sequence divergence between these two species on the end of Chromosome 20 could be over-represented, whether or not there was a genuine expression difference among the alleles.

By separately mapping reads from SS pheromone glands to alleles found in SS individuals, and mapping reads from VV pheromone glands to alleles found in VV individuals (which are mostly from the *subflexa* genome except for the *virescens*-derived end of Chromosome 20), we found that *Est1*, *LipX*, and *LipZ* are the only genes with a significant over-expression in VV pheromone glands compared to SS (Supplementary Table S5). *LipZ* is the most differentially expressed gene between VV and SS, which is mostly due to the fact that it has a very low expression in the SS pheromone glands. As *LipY* is absent from the *H. virescens* genome, its expression ratio is zero in SS individuals. Thus, the two pairs of tandemly repeated genes (*LipX*-*Z* and *Est1*-*2*) that had surfaced in the original analysis are most probably involved in the difference in the amount of acetates between the SS and VV female pheromones.

### In vivo functional characterization of candidate genes

**Fig. 4:**
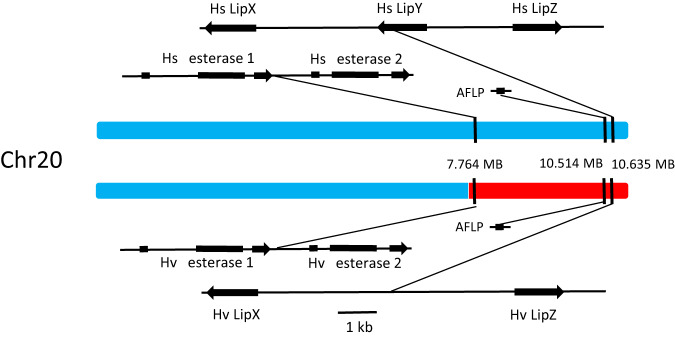
Lipase and esterase genes in *H. subflexa* and the introgressed portion of Chromosome 20 from *H. virescens*. Diagram of the S and V allele of chromosome 20 from DD23 *H. subflexa* displaying the position of *LipX*, *LipY*, *LipZ*, *Est1*, and *Est2* as well as the *H. virescens* introgression in the V allele (in red).

**Fig. 5:**
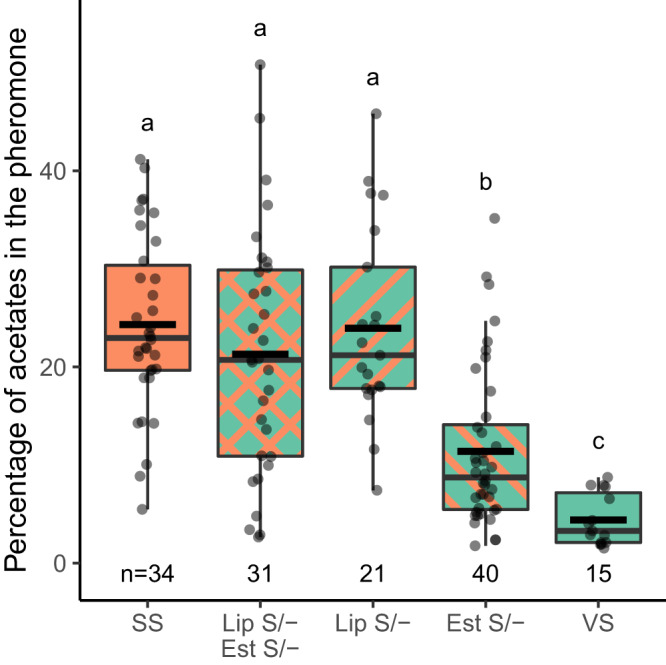
Effect of the different knockouts on the acetate levels. Boxplots of the relative percentage of the sum of the three acetates, in female pheromone glands. SS individuals (plain orange) are wild type, VS individuals (plain green) carry one copy of *H. virescens* Chr20-QTL. *Est* s/- (green with top left to down right orange stripes) stands for VS individuals for which the V alleles of *Est1* and *Est2* have been knocked-out, *Lip* s/- (green with down left to top right orange stripes) are also VS individuals but this time the V alleles of *LipX* and *LipZ* have been knocked out. Finally, *Lip* s/- *Est* s/- (green with double orange stripes) stands for VS females that have the V alleles of the four aforementioned genes knocked-out. Boxplots are built similarly as in Fig. 2. Letters represent group of statistical similarity based on Welch one-way ANOVA followed by Games-Howell post-hoc test (exact *p* values in Supplementary Table S6). Source data are provided as a Source data file.

To determine the role of the identified candidate genes differentially expressed between SS and VV individuals, we performed loss-of-function studies by generating mutant DD23 lines using CRISPR/Cas9, where we knocked out the *H. virescens* alleles of *LipX*, *LipZ*, *Est1*, and *Est2* in the introgressed segment of Chr20 (Fig. 4). To avoid functional compensation between closely related enzymes, we targeted multiple genes at once in each knock-out line. In the esterase-only knock-out line, we found intermediate percentages of acetate levels compared to VS and SS (Games-Howell post-hoc test, *p* < 0.05, Supplementary Table S6; Fig. 5). A similar pattern was observed when we log-contrasted the total amount of acetate esters to tetradecanal (14:Ald) to break data interdependency (see “Methods”; Supplementary Fig. S2k). In the lipase-only knock-out line, we found similar acetate levels as in SS and significantly lower acetate levels in VS females (Games-Howell post-hoc test, *p* < 0.05, Supplementary Table S6). We observed similar results when both the lipases and esterases were knocked out (Fig. 5 and Supplementary Fig. S2k).

We did not find significant differences between the five genotypes in the total amount of pheromone produced, except between VS and the esterase knock-out line, which is most probably due to one VS outlier (Supplementary Fig. S2a). In comparing differences in the three other components that affect male response (Z-9-hexadecanal (Z9-16:Ald), Z-11-hexadecanal (Z11-16:Ald), and Z-11-hexadecanol (Z11-16:OH)), we found that the lipase knock-out line as well as the four-genes knock-out line had similar log-contrasted levels of Z9-16:Ald, Z11-16:Ald and Z11-16:OH as SS females, but significantly lower levels than VS females (Supplementary Fig. S2l–o). The esterase knock-out line had similar levels of the three compounds as VS and SS females apart from a significantly higher levels of log-contrasted Z11-16:OH compared to SS females (Supplementary Fig. S2l–o). When comparing relative percentages, 14:Ald levels were similar between VS and esterase knockout females and significantly lower compared to SS females. Double and lipase only knockouts lines had similar levels compared to SS. All lines had similar levels of Z9-16:Ald (Supplementary Fig. S2h). VS and esterase knock-out line had significantly higher proportion of Z11-16:Ald in comparison to the other genotypes (Supplementary Fig. S2i). The SS and lipases knock-out lines had a significantly smaller relative percentage of Z11-16:OH in their pheromone compared to VS. The percentage of Z11-16:OH in the double and esterase-only knock-out lines were not significantly different from those in the VS lines (Supplementary Fig. S2j).

### Docking of pheromone compounds in the candidate enzymes

**Fig. 6:**
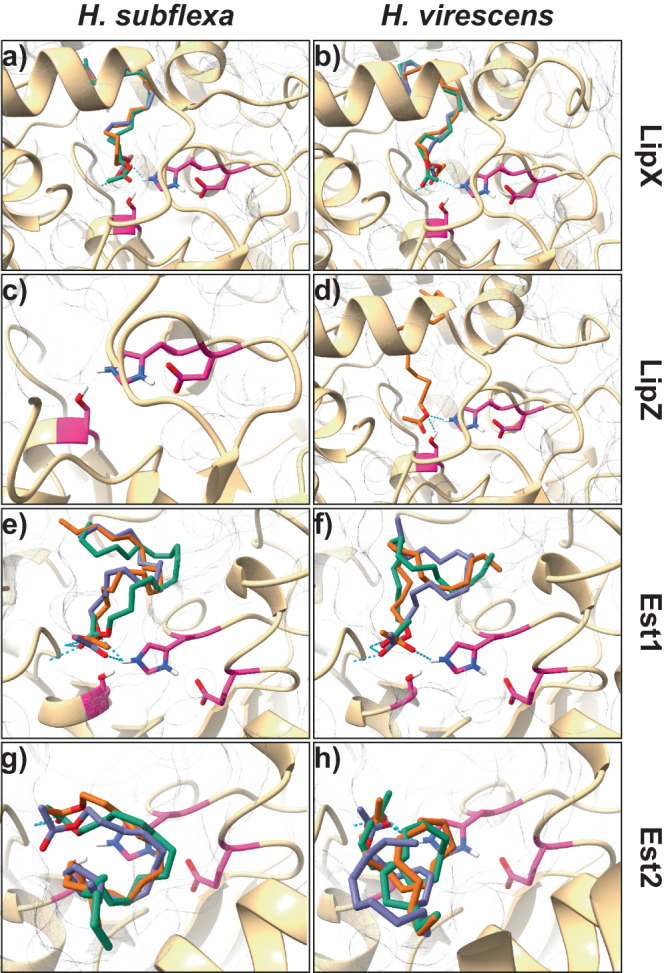
Docking of pheromone acetate compounds in candidate enzymes. Models of the docking of Z7-16:OAc (display in violet), Z9-16:OAc (in turquoise green) and Z11-16:OAc (in orange) into the predicted 3D structure of *H. subflexa* (**a**, **c**, **e**, **g**) and *H. virescens* (**b**, **d**, **f**, **h**) isoforms of LipX (**a**, **b**), LipZ (**c**, **d**), Est1 (**e**, **f**), and Est2 (**g**, **h**). Residues that are expected to belong to the catalytic triads based on homology are shown as ball and sticks and colored in magenta. Displayed oxygen, hydrogen, nitrogen, and sulfur atoms are respectively colored in red, white, blue, and yellow. Predicted hydrogen bonds are displayed as dashed cyan lines. Surface of the protein is shown as a gray mesh. Code and input data used to obtain this figure have been deposited on figshare (10.6084/m9.figshare.22656718 and 10.6084/m9.figshare.24306418).

To explore the feasibility of the hydrolysis of the three *H. subflexa* acetates by the candidate lipases and esterases, we used in silico molecular modeling and docking studies. For comparison, we first used *Spodoptera littoralis* SlitCXE7, SlitCXE10 and three of their experimentally verified substrates, which include two acetate pheromone compounds^35,36^. We observed docking models of the SlitCXE7 and CXE10 substrates without steric clashes and with the carbonyl carbon within 4.5 Å of the catalytic serine of the enzymes (Supplementary Fig. S3). Both enzymes had been shown to hydrolyze acetate esters by heterologous expression in previous studies^35,36^. Using the same modeling protocol, we observed that *H. subflexa* and *H. virescens* isoforms of LipX, Est1, and Est2 can accommodate substrates Z-7-hexadecenyl-acetate (Z7-16:OAc), Z-9-hexadecenyl acetate (Z9-16:OAc), and Z-11-hexadecenyl acetate (Z11-16:OAc) in their binding pockets (Fig. 6a, b, e–h; Supplementary Fig. S4a, b, e–h). For LipZ, we only found docking models of Z11-16:OAc, not of Z7 and Z9-16:OAc in HvirLipZ (Fig. 6c, d; Supplementary Fig. S4c, d). For HsubLipZ, no docking models of tested acetate pheromone compounds were found. Overall, the binding pockets of LipX and LipZ appear less permissive than the binding pockets of Est1 and Est2 (Fig. 6, Supplementary Fig. S4a–d).

## Discussion

By conducting RNAseq on female sex pheromone glands from two closely related species with acetate esters (*H. subflexa*) or without them (*H. virescens*) as well as acetate QTL introgression lines (VV and SS), we identified esterases and lipase genes involved in moth sex pheromone acetate levels. Introgressing this QTL into the *H. subflexa* genomic background provided the opportunity to examine the role of acetate hydrolysis in affecting the final amount of acetates in female pheromone glands. This question cannot be addressed by studying female *H. virescens*, unless synthetic acetates are applied to the gland, as was done by Teal and Tumlinson^37^. The added acetate esters were hydrolyzed, and Teal and Tumlinson commented on finding this apparently unnecessary hydrolytic activity in the gland. “The presence of the esterase in the pheromone gland of *H. virescens* is an enigma, because none of the acetates […] have been identified from extracts”^37^. No attempts were made to identify the responsible enzymes in these early studies. However, our introgression of a portion of *H. virescens* Chromosome 20 containing esterase and lipase genes provides the opportunity to test hydrolytic function in a pheromone gland that naturally produces acetates, rather than artificially adding them.

The results from CRISPR/Cas9 knockouts prove that hydrolysis will decrease the final amount of acetate esters. Our experimental design does not reveal the contribution of individual enzymes, because to avoid a possible compensatory effect we targeted both of the esterases and/or both lipases at once. We reasoned that two CRISPR/Cas9 experiments could be completed more easily than four, and in fact the low success rate as shown by the need to repeat the attempt three times justifies this. Our results show that knocking out the esterases alone has less effect on the acetate levels than knocking out the lipases (see Fig. 5 and S2), making the lipases the more likely candidates, but since knocking out the esterases alone significantly increased the acetate levels (Fig. 5), we cannot dismiss the role of esterases. Functional characterization by heterologous expression of the enzymes will be needed to confirm their enzymatic activity as well as their annotation as lipases and esterases.

We find it important to note that we previously found a second major QTL underlying acetate variation^30^, which indicates that additional and possibly interacting enzymes are involved in acetate biosynthesis and/or degradation. Unfortunately, even after 10 years of effort, we have never been able to introgress this second QTL region from *H. virescens* into *H. subflexa* and we are currently exploring different approaches to investigate the presence of potential hydrolyzing genes in this QTL and possible interaction effects.

Carboxylesterases have been extensively studied for their potential role in degradation of acetate esters in male antennae^38–43^. Thus, moth pheromone compounds being degraded by an esterase is not surprising. However, it has never, to our knowledge, been shown that an esterase can have an effect on the composition of a moth sex pheromone. Lipases have rarely been studied in relation to moth pheromone metabolism. The function of lipases has been mainly considered in digestion, following work in vertebrates. However, there is some previous evidence supporting that lipases can be involved in moth pheromone biosynthesis, although not regarding the hydrolysis of the terminal compounds. In moths, the hormone PBAN, which triggers pheromone production in female pheromone glands^44,45^, also activates lipase activity in the pheromone gland in some Apoditrysia (Lepidoptera) species^46,47^. Du et al. highlighted the expression of some lipases, including three acidic lipases, in the female pheromone glands of *Bombyx mori*^48^. Using RNAi, they found that two of the acidic lipases increase the proportion of bombykol in the female pheromone^48^. Thus, there are precedents for the involvement of esterases and lipases in Lepidoptera sex pheromone metabolism.

To our knowledge, no publication on the pheromone composition of *H. virescens* females has ever reported finding acetate esters. Previous interspecific crosses of *H. virescens* and *H. subflexa* have shown that F1 hybrid females produce no or greatly reduced amounts of acetates compared to *H. subflexa* females^49,50^. Absence of the gene(s) encoding acetyltransferases from the genome of *H. virescens* and their presence only in the genome of *H. subflexa* cannot be the explanation, because acetate esters have been found in the hairpencils of male *H. virescens*^28^, as well as on *H. virescens* legs^29^. Therefore, *H. virescens* must possess one or more acetyltransferase genes, which are expressed in the male hairpencils but not the female pheromone gland. The acetyltransferase gene(s) of *H. subflexa* are likewise expressed in the pheromone glands of hybrids, as well as DD23 females.

Teal and Tumlinson suggested that “the esterases present in the gland of females of *H. virescens* converts the acetates into alcohols as rapidly as they are produced”^37^. They hypothesized that the hydrolytic activity in *H. virescens* “may act as fail-safe mechanism which converts any acetate which might enter the cuticle to the alcohol”^51^. Another possibility is that females hydrolyze acetate esters from the hairpencils of *H. virescens* males^28^, which are transferred to the female during mating and greatly reduce attraction of subsequently encountered *H. virescens* males as well as her reproductive output^52^. An hydrolytic activity in and around the pheromone gland would limit the impact of this male chemical mate guarding, which would resemble the degradation by *Drosophila melanogaster* females of sex and aggregation pheromone cis-vaccenyl acetate^53,54^. Thus, the expression in the pheromone gland and the acetate esters hydrolysis activity of HvirLipX, LipZ, Est1, and/or Est2 may have evolved in the trajectory of *H. virescens* as a “fail-safe” and/or “anti-mate guarding” function.

An alternative scenario is that the expression patterns and enzymatic activities of these lipases and esterases trace back to an acetate-producing ancestor. Since in moths acetates are the most commonly used pheromone compounds^17^, and most species in the Noctuidae contain acetates in their sex pheromone blends^6^, the absence of acetates in the pheromone gland is likely to be a derived state. Unveiling the role of these lipase and esterase genes in *H. virescens* mate recognition, and potentially extending our insights across Lepidoptera, presents a compelling avenue of research opened by the work presented here. To determine how these enzymes are involved in the fine tuning of acetate levels in the pheromone blend, an exploration of Lepidoptera genomes and pheromone gland transcriptomes is needed, aiming to elucidate the evolutionary history of *LipX*, *LipZ*, *Est1*, and *Est2* orthologues along with their expression patterns in other species. Complemented by functional studies on both extant and reconstructed ancestral genes^55^, this approach should provide valuable insights into the role of these lipase and esterase genes in regulating Lepidoptera sex pheromone blend composition and their evolution.

Our study underlines the effectiveness of introgressing a major QTL into the genomic background of a related species for transcriptome and loss-of-function analyses, to identify new genes underlying a phenotype. Through these analyses, we revealed that lipases and carboxylesterases are involved in moth sex pheromone metabolism, specifically in the degradation of acetate esters. As the role of these enzyme families in moth sex pheromone biosynthesis was previously unexpected, this paves the way for new biochemical and molecular analyses to gain insights in the specific functions of these enzymes in moth sex pheromone biosynthesis. In addition, the attempts to identify acetyltransferases must continue, because only after identification of the relevant genes will it be possible to explain how *H. subflexa* evolved this very effective reproductive isolation mechanism from *H. virescens*. Our study also highlights that pheromone degradation needs to be considered as an important mechanism in the evolution of moth sex pheromones.

## Methods

### Insect rearing

The genus name *Heliothis* was recently changed to *Chloridea* for *subflexa* and *virescens* only^24^ but we retain *Heliothis* for continuity with the extensive prior literature. Animals were kept at 25 °C and 60% relative humidity with a reversed 14 h:10 h day-night cycle (lights off at 11.00, on at 21.00). *H*. subflexa larvae were maintained on wheat germ/soy flour-based diet (BioServ Inc., Newark, DE, USA). Adult moths were provided with a cotton roll soaked in 10% sugar water. The so-called DD23 strain was generated by hybridizing *H. virescens* and *H. subflexa*, followed by continuous backcrossing to *H. subflexa*, as described in detail previously^30,31,33^. In short, we generated multiple backcross lines (in order to avoid brother-sister matings which have detrimental effects in Lepidoptera^56^), to conduct intercrosses. This resulted in the introgression of a terminal part of HvirChr20-QTL from *H. virescens* into a complete *H. subflexa* genomic background (Supplementary Fig. S1), which led to significantly lower acetate levels in the pheromone blend (Fig. 2)^31^. In this study, we used animals homozygous (VV), heterozygous (VS), and wild-type (SS) for this QTL. As this QTL is dominant, i.e., one V-copy is enough to reduce the acetate levels^31^ (Fig. 2), we used VS females in the loss-of-function analyses. Distinction between the three genotypes was made through PCR amplification of marker sequences where an EcoRI restriction site is present in V-allele and absent in the S-allele.

Maintaining the introgressed Chromosome 20 in the *H. subflexa* background over several generations required special attention. A strain of homozygous VV cannot be maintained for very long, due to inbreeding effects^56^ caused by homozygosity for the *virescens*-derived segment of Chromosome 20 as well as the *subflexa*-derived segment that has become fixed. A population segregating the three types VV, VS, and SS can be maintained for a few generations, but the frequency of the V type constantly declines due to its lower fitness. To prevent its loss, adults are screened (by PCR of DNA isolated from a single leg) before mating or mated pairs are screened after mating, and fewer SS individuals are taken to the next generation. The first round of injections used eggs laid by females in VV x VV single-pair matings. Subsequent rounds used individuals from a segregating population crossed to *H. subflexa*, therefore SS progeny had to be screened out because they would be uninformative for knockouts of the *virescens* alleles. Occasionally the overall fitness (larval survival, pupation success, adult fertility) of the segregating population was so low that it had to be outcrossed to *H. subflexa* (which reduced the frequency of the introgressed chromosome to half its original value) and then the progeny screened so that the introgressed chromosome could be increased in frequency once again. Over the course of several years, the segregating population was lost in Amsterdam and had to be replaced from Jena, and vice-versa.

### Pheromone extraction

For all pheromone analyses in this study, we used 2- to 4-day-old virgin female moths. Females, whose glands were used in the RNAseq and the real-time quantitative PCR, were injected with pheromone biosynthesis activating neuropeptide (PBAN) 1–2 h before gland extraction to stimulate pheromone production, as previously done^7,30,44^. To avoid possible side-effects of PBAN injections in our subsequent functional analysis experiments, pheromone extractions were performed only on uninjected calling females, 3–4 h into scotophase. Pheromone gland extractions were conducted as described in detail previously^27^, and summarized here. Pheromone glands were cut with microdissection scissors, soaked for 20–30 min in 50 µL of hexane containing 200 ng of pentadecane as an internal standard, and stored at −20C until analysis. For analysis, samples were reduced to 1–2 µL under a gentle stream of N_2_ and injected into a splitless inlet of a HP6890 gas chromatograph coupled with a high-resolution polar capillary column [DB-WAXetr (extended temperature range); 30 m 90.25 mm 90.5 Mm] and a flame ionization detector (FID). The area under the pheromone peak was estimated using integration software implemented in Agilent ChemStation (version B.04.03). The absolute amounts (in ng) of the eleven identified components of the *H. subflexa* sex pheromone blend (14:Ald, Z7-14:Ald, 16:Ald, Z7-16:Ald, Z9-16:Ald, Z11-16:Ald, Z7-16:OAc, Z9-16:OAc, Z11-16:OAc, 16:OH, and Z11-16:OH; see ref. ^57^) were calculated relative to our 200 ng pentadecane internal standard and divided by the total amount to get the relative amount. To break data interdependency and to correct for individual variation in the amount of pheromone produced that play a role in male response (see ref. ^58^), the total amount of the three acetate-esters, Z9-16:Ald, Z11-16:Ald, and Z11-16:OH were divided by the amount of 14:Ald. We chose 14:Ald as the divisor, because it does not affect male response behavior^59^ and was present in comparable amounts in all extracts, except SS and the esterase knock-out line (Supplementary Fig. S2.b and Supplementary Table S6). Log transformation was performed when this corrected non-normality of residuals. Samples with less than a total pheromone amount of 40 ng were excluded, because the ratios of the minor compounds could not be accurately quantified in the chromatograms in these samples.

### DD23 strain pheromone gland transcriptome assembly

For our transcriptome analysis, we extracted RNA from the pheromone glands of 10 females, five of VV genotype and five of SS, using Ambion™ TRIzol™ Reagent (Thermo Fisher Scientific, Waltham, Massachusetts, USA) and Direct-zol™ RNA MiniPrep Kit (Zymo Research, Irvine, California, USA). Previously the pheromones from each of the glands had been extracted with hexane as described above. The RNA was sequenced using Illumina HiSeq2500 by BaseClear (Leiden, The Netherlands). Raw reads which were used to assemble the pheromone gland transcriptome were deposited in the NCBI Sequence Read Archive under the bioproject number PRJNA493752. Resulting reads were trimmed of adapters and for a Phred quality score of 28 or more using Trimmomatic^60^. We then removed ribosomal reads using Ribopicker^61^. We also removed reads without a mate pair. The remaining reads were normalized and de novo assembled using Trinity^62^. We then aligned the Trinity output with the R1 reads used for assembly with Bowtie2^63,64^ and removed all contigs matching less than five reads. The resulting contigs will be further referred to as the DD23 pheromone gland unfiltered transcriptome. To improve the quality of the transcriptome, we sequentially used three tools as described in ref. ^65^ and summarized here. We first filtered out the contigs without an ORF of 100 amino or more using Transdecoder (http://transdecoder.github.io/). We then used RSEM^66^ to remove all contigs with an TPM expression value lesser than one. Finally, we clustered and removed redundant contigs using CD-HIT EST (90% similarity threshold and word size of eight)^34,67^. To assess the quality of this reference transcriptome, we used BUSCO^68^ and Transrate^69^.

### Differential expression analysis and differentially expressed transcripts annotation

Differences in expression levels were explored by aligning the reads used for the assembly onto the reference transcriptome, using RSEM^66^, after which we conducted differential expression analysis, using DESeq2^70^ and edgeR^71^ tools. We selected contigs differentially expressed between SS and VV genotypes by filtering contigs with: a ±2 log2 fold change in expression value (Deseq2) and an associated adjusted *P*-value (from DEseq2 and edgeR) of 0.05 or lower. We annotated these differentially expressed transcripts by blasting them against the nr NCBI database^72^, using blastx^73^, and attributed GO terms using Blast2GO^74^.

Subsequently, an unbiased expression analysis was conducted by mapping SS reads to contigs derived from genes present in SS individuals, and mapping VV reads to contigs derived from genes present in VV individuals (Supplementary Data 1). We used the same analytic workflow described above.

### Sequencing of candidate transcripts and quantitative real-time PCR

To confirm in silico differential expression results, we first had to confirm the sequences of the candidate transcripts, which we did by extracting RNA from *H. virescens* and *H. subflexa* female pheromone glands; from different individuals than those that provided the glands for the expression analysis described above. RNA was reverse-transcribed using the Verso cDNA Synthesis Kit (Thermo Fisher Scientific), the candidate gene transcripts were amplified using DreamTaq™ DNA Polymerase (Thermo Fisher Scientific) and primers were designed to amplify an approximately 1000 nucleotide region from cDNA from *H. virescens* and *H. subflexa* pheromone glands (see Supplementary Table S7 and Supplementary Figs. S5–S8). Amplicons were sequenced using Sanger sequencing by Marcogen (Amsterdam, the Netherlands). From these sequences, we designed primers for real-time PCR to get approximately 100 bp amplicons from the alleles of both species for each target gene.

To assess the in vivo expression of the candidate genes, we extracted RNA as described above from 5-6 pheromone-extracted pheromone glands of *H. subflexa*, *H. virescens*, SS and VV females. We used 20 µl reaction of 5x HOT FIREPol® EvaGreen® qPCR Mix Plus (ROX) (Solis BioDyne, Tartu, Estonia), 2 ng of cDNA and 1 µM of primer couples for the target genes and used RPS18 as a reference gene. For primers used, see Supplementary Table S7 and Supplementary Figs. S5–S8. All biological replicates were also technically replicated. qPCR reactions and measurements were made using an Applied Biosystem 7500 Real-Time PCR System (Thermo Fisher Scientific).

### Knock-out of the candidate genes using CRISPR/Cas9

To functionally characterize the candidate genes discovered in the previous steps, CRISPR/Cas9 knock-out experiments were performed as follows. The IDT system for CRISPR/Cas9 was used (Integrated DNA Technologies). Gene-specific crRNAs are annealed with tracrRNAs and incubated with Cas9 enzyme to form ribonucleotide particles for injection into eggs. Gene-specific guide RNAs were designed for the two esterases (VV*Est1*-T1 and VV*Est1*-T2 for est1, VV*Est2*-T1 and VV*Est2*-T2 for *Est2*) and the two lipases (VV*LipX*-T1 and VV*LipX*-T2 for *LipX*, VV*LipZ*-T1 and VV*LipZ*-T2 for *LipZ*, all sequences are in Supplementary Table S7 and Supplementary Figs. S5–S8). First, VV eggs were injected with *Est1*-*Est2* guide RNA to knock out the two esterases. In a second step, VV and VV-*Est1*-*Est2*-ko eggs were injected with *LipX*-*LipZ* guide RNAs to obtain respectively a line with only the two lipase knock-outs and another line with the four gene knock-outs (*Est1*, *Est2*, *LipX*, and *LipZ*). All eggs were from 30 min to 1 h old and micro-injected using a Femtojet (Eppendorf) with a solution previously loaded in home-made glass needles. The solution was prepared by first dissolving each of the four scRNAs (Supplementary Table S7 and Supplementary Figs. S5–S8) in 1 nmol of tracrRNA to get a final concentration of 50 μM, after which 200 pmol of this scRNA+tracrRNA mix was combined with 100 pmol of IDT Alt-R Cas9.

Injection needles were filled with about 2 μl of injection solution. For esterase knockouts, the following ingredients were combined: 100 pmol of IDT Alt-R Cas9 enzyme in 1.6 μl, 150 pmol total of the guide RNAs consisting of equal parts of crRNAs for VV*Est1*-T1, VV*Est1*-T2, VV*Est2*-T1, and VV*Est2*-T2 in 3.0 μl, 50 pmol of guide RNA targetting the *scarlet* gene affecting larval pigmentation in 1.0 μl, and 34.4 μl H_2_O. The *scarlet* guide RNA was omitted for injections at later dates and the amount of guide RNAs was increased. Thus, the molar ratio of guide RNAs to Cas9 enzyme was 2:1. Guide RNAs were made by combining crRNAs and tracrRNAs in equimolar ratios in duplex buffer, heated at 94 C for 2 min, and allowed to cool at room temperature. Lipase injections were the same except for the guide RNAs VVLipX-T1, VVLipX-T2, VVLipZ-T1, and VVLipZ-T2.

The gauze with the injected embryos was kept in Petri-dishes at lab temperature (20 °C) in an air-tight chamber provided with a damp sponge and checked daily. After three days, newly hatched neonates were separated in individual cups with wheat germ/soy flour-based diet (BioServ Inc., Newark, DE, USA). For some egg cloths, eggs were left uninjected to verify fertility later; these were marked on the photograph and removed with sticky tape two days later when eyespots developed so that larvae emerging from them would not cannibalize the injected eggs, which develop more slowly.

A scheme displaying the crosses and details on the CRISPR experiments can be seen in Supplementary Fig. S9. In 2019, 404 eggs on 25 different cloths were injected (average 16.16, s.d. 12.1, minimum 4, maximum 54). There was a total of 359 eggs injected with esterase guide RNAs and 45 with lipase RNAs. Fifty eggs survived the injection (12% survivorship) and neonates emerged and were collected into vials. Surviving adults were outcrossed to *H. subflexa*, and although fertility was low and many surviving adults did not produce progeny, eventually one haplotype with deletions in both esterase genes was recovered in subsequent generations. No lipase mutant haplotypes were recovered in 2019. Screening primers were specific to the *H. virescens* gene and would not amplify the *H. subflexa* gene, therefore the introgressed chromosome carrying the *virescens* mutated genes could be identified by screening, and separated from any off-target mutations that might have occurred in the *subflexa* homolog.

To identify mutations in the genes, we performed DNA extractions on newly emerged adults by soaking one foreleg in 30 µl of 10% Chelex and 2.5 µl of Proteinase K during 3 h at 56 °C, then 8 min at 98 °C and by finally freezing the mixture before usage (adapted protocol from BioRad). Distinction between the genotypes was made through PCR amplification with gene-specific primers: VV*Est1*-scr-Fd and VV*Est1*-scr-R for *Est1*, VV*Est2*-scr-F and VV*Est2*-scr-R for *Est2*, VV*LipX*-scr-Fd and VV*LipX*-scr-R for *LipX*, and VV*LipZ*-scr-F and VV*LipZ*-scr-R for *LipZ*; all sequences are in Supplementary Table S7 and Supplementary Figs. S5–S8. Amplification followed the Thermo Fisher Phire Hot start II 3-step protocol with 33–35 cycles of 30 s (10 s for each step) and an annealing temperature of 60 degrees. The reactions were performed in 10 μl with 2 μl of DNA, 2 μl PCR Buffer, 2 μl of 1 mM dNTPs, 2 μM for each primer, and 0.1 μl of Phire hot start II polymerase. PCR products were analyzed by electrophoresis on 2% agarose gels in TAE buffer (40 mM Tris-acetate, 2 mM EDTA), and the resulting bands were visualized with midori green (Biozym). *Est1*-*Est2* mutants were cloned and sequenced at the Max Planck Institute for Chemical Ecology and *LipX*-*LipZ* mutants were cloned and then sequenced by Macrogen EZ-seq (Amsterdam, Netherlands) (Supplementary Table S7 and Supplementary Figs. S5–S8). Individuals with mutations were subsequently outcrossed with *H. subflexa* to prevent the negative consequences of inbreeding and to keep the introgressed chromosome on a *subflexa* background, and their progeny was reared as above. Every generation, this procedure was repeated to genotype all adults, while pheromone gland extractions were performed on a subset of females. *Est*-KO females were collected from the first generation after outcrossing. *Est*-*Lip*-KO and *Lip*-KO females were from the first three generations after outcrossing.

### Docking of acetate pheromone compounds in candidate enzymes

We assessed whether predicted 3D structures of the candidate enzymes could accommodate the acetate pheromone compounds produced by *H. subflexa* females. Structure of Z7, Z9, and Z11-16:OAc were obtained from Pherobase^6^ in PBO format and converted into PDBQT using OpenBabel^75^. We removed potential signal peptides from the sequences of the *H. subflexa* and *H. virescens* alleles of *LipX*, *LipZ*, *Est1*, and *Est2* using PrediSi^76^. 3D structure of the sequences was predicted using ColabFold^77^. Residues in the catalytic triad were identified by alignment with previously-annotated lipases and esterases (Supplementary Figs. S10–11). Ligand and enzyme files obtained were prepared for the docking software using AutoDockTools, part of MGLTools v1.5.7^78^. 50 Ligand binding models were generated for each enzyme using Vina v1.2.3^79^ and 10 exhaustiveness. Using Chimera X^80^, we computed potential hydrogen bonds and steric clashes between the ligands and the enzymes and measured the distance between the oxygen of the catalytic serine and the carbon of the carbonyl group of the ligands. We display the ligand binding models that have the lowest affinity, no steric clashes and the carbonyl carbon to serine oxygen distance less than 4.5 Å. As a positive control, we confirmed that with this method we could observe the binding of *S. littoralis* acetate pheromone in SlitCXE7 and SlitCXE10 (Supplementary Fig. S3) consistently with the experimental study of Durand et al.^35,36^.

### Data analysis

Data and statistical analysis were performed using R Studio version 1.0.136 with R version 3.3.2. Bartlett’s test (function *bartlett.test*, package s*tats*) was used to test for homogeneity of variance of the difference between the Ct observed for the reference and the target genes in the qPCR data, pheromone data of DD23 line and of the five genotypes in the loss-of-function analysis. Homogeneity of the means of the same values was tested using a Welch one-way ANOVA (function *oneway.test*, package *stats*). A Tukey or a Games-Howell post-hoc test (custom R script, http://aoki2.si.gunma-u.ac.jp/R/src/tukey.R) was used for the groups with homogeneous or non-homogenous variance, respectively. Quantitative PCR results were expressed in target copy number relative to 1000 copies of RPS18, similarly as Groot et al.^21^.

### Reporting summary

Further information on research design is available in the Nature Portfolio Reporting Summary linked to this article.

### Supplementary information

Supplementary Information

Reporting Summary

Description of Additional Supplementary Files

Supplementary Data 1

### Source data

Source Data

## Data Availability

The *H. subflexa* and *H. virescens* lab strains used in this study are available upon request to A. T. Groot (A.T.Groot@uva.nl). The raw reads used to assembled the *H. subfelxa* DD23 pheromone gland transcriptome data generated in this study have been deposited in the NCBI database under accession code PRJNA493752. The sequences resulting from genomic DNA Sanger sequencing generated in this study have been deposited in the NCBI database under accession code OK556469, OK556470, OK556471, OK556472, OK556473, OK556474, OK556475, and OK556476. The pheromone quantification and real-time PCR data generated in this study are available in figshare under the 10.6084/m9.figshare.22656718. The Input data for in sillico docking experiment analysis data generated in this study have been deposited in figshare under the 10.6084/m9.figshare.24306424. Source data are provided as a Source data file. Source data are provided with this paper.

## Code availability

Code for in sillico docking experiment analysis used in this study has been deposited in figshare under the 10.6084/m9.figshare.24306418.
