# Supplementary material for: Lipases and carboxylesterases affect moth sex pheromone compounds involved in interspecific mate recognition
Source: Nat Commun. 2023 Nov 18;14:7505. doi: 10.1038/s41467-023-43100-w (PMC10657362; doi:10.1038/s41467-023-43100-w)
Supplement: Supplementary file 1 — Supplementary Information [file 41467_2023_43100_MOESM1_ESM.pdf]

Supplementary Information

Supplementary Fig. S1: Karyotype of DD23 SS and VV individuals

Scheme of the karyotype of DD23 SS wild-type *H. subflexa* individuals and VV individuals with the introgressed region of *H. virescens* alleles (violet) in Chr20.

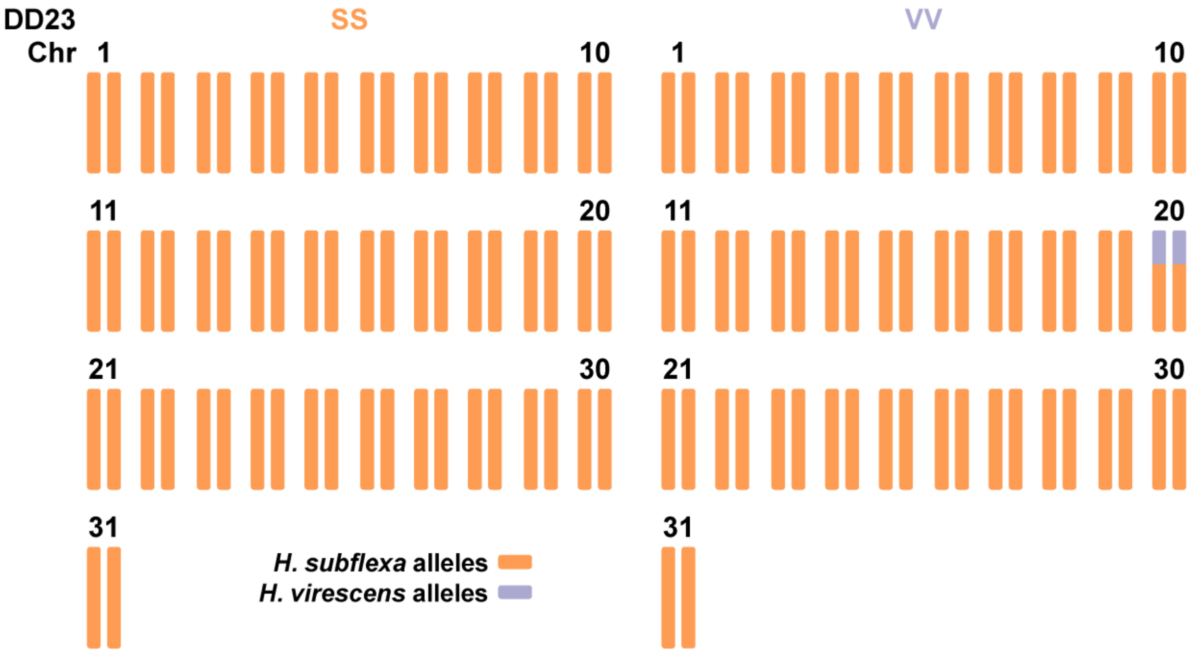

Supplementary Fig. S2: Effect of the different knock-outs on the pheromone composition

Boxplots of: a) the total amount of pheromone extracted per female in ng, **b**-**f**) the quantity (ng/female) of different components of the pheromone blend, **g**-**k**) the percentage of different compounds in the pheromone relative the total amount of pheromone, **l**-**o**) the percentage of different components of the pheromone blend divided by the quantity of 14:Ald and log transformed. Each small grey dot represents an individual data-point. SS individuals (plain orange) are wild type, VS individuals (plain green) carry one copy of *H. virescens* Chr20-QTL, Est s/- (green with top left to down right orange stripes) stands for VS individuals for which Est1 and Est2 have been knocked-out, Lip s/- (green with down left to top right orange stripes) are also VS individuals but this time LipX and LipZ are not functional. Finally, Lip s/- Est s/- (green with double orange stripes) stands for VS females that have the four aforementioned genes knocked-out. Boxplots are built similarly as in Fig. 2. Letters represent group of statistical similarity based on Welch one-way ANOVA followed by Turkey or Games-Howell post-hoc test (exact p-values in Supplementary Table S3).

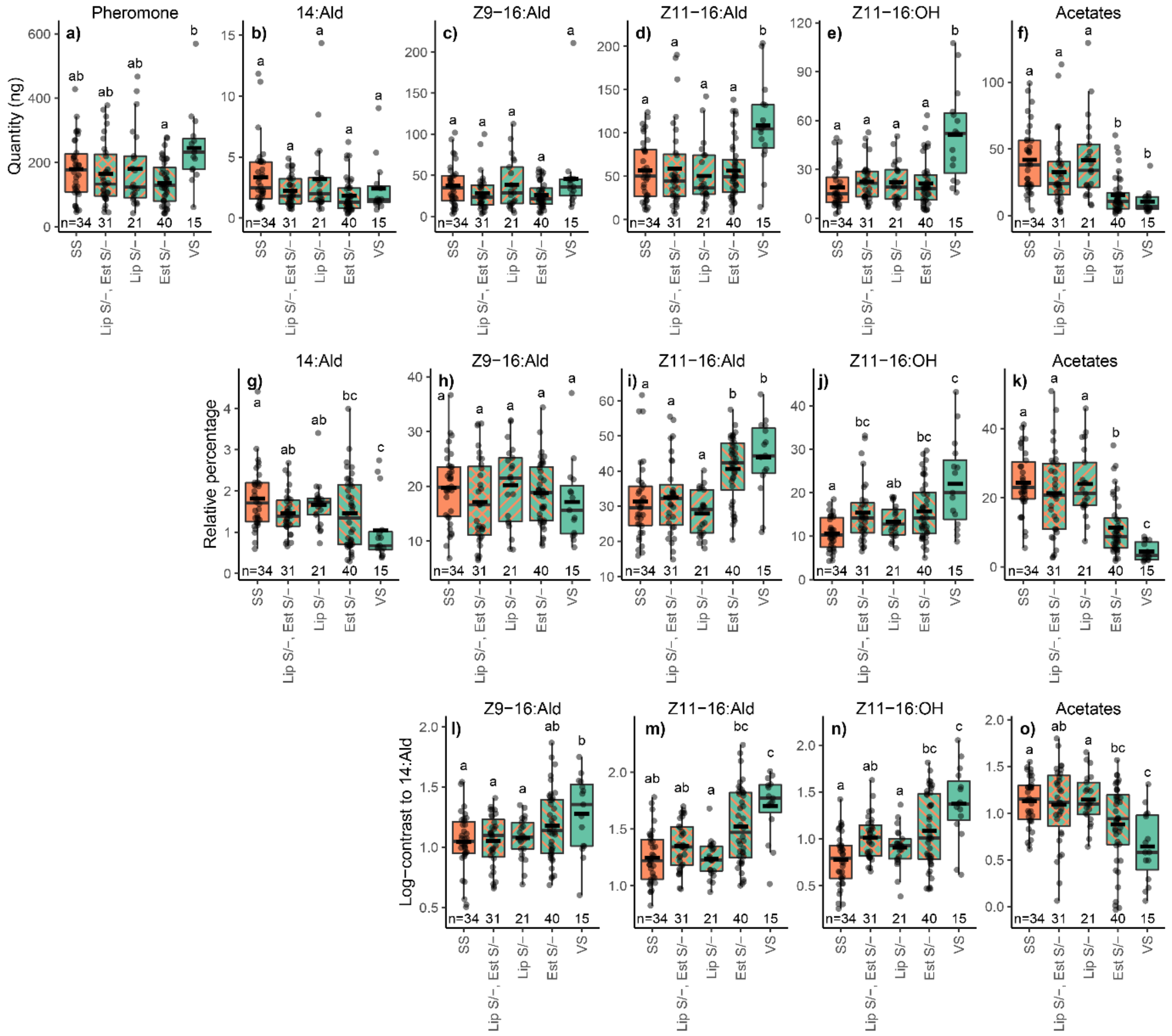

Supplementary Fig. S3: Docking of acetate compounds in SlitCXEs

Docking of (Z)3-hexenyl acetate (display in violet), Z9E11-14:OAc (in turquoise green) and Z9E12-14:OAc (in orange) into the predicted 3D structure of *S. littoralis* CXE7 (**a**, **b**) and CXE10 (**c**, **d**) Residues expected to belong to the catalytic triads based on homology are shown as ball and sticks and colored in magenta. Displayed oxygen, hydrogen, nitrogen and sulfur atoms are respectively colored in red, white, blue and yellow. Predicted hydrogen bonds are display as dashed cyan lines. In **c** and **d**.

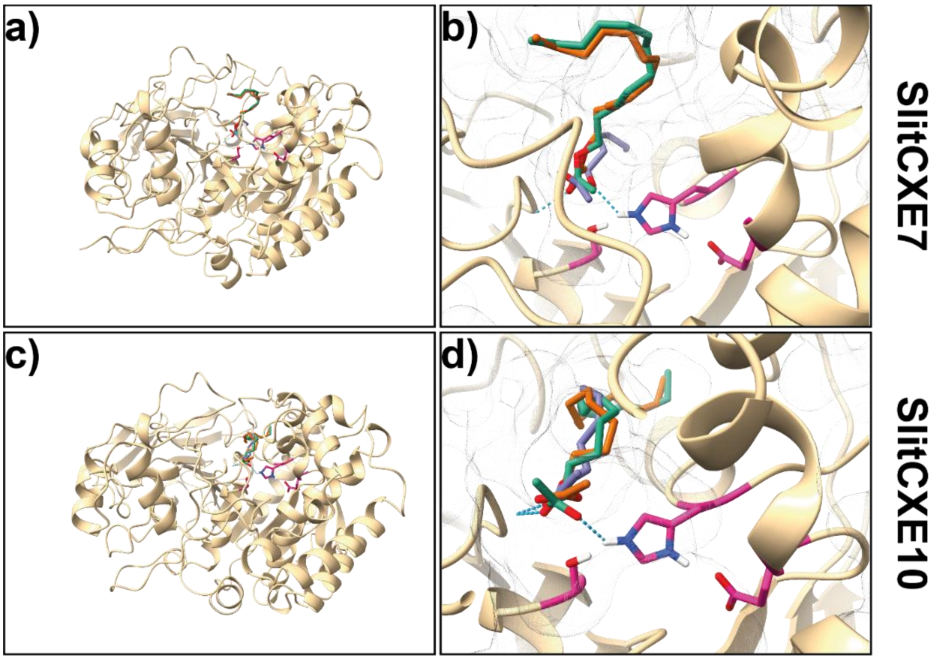

Supplementary Fig. S4: Zoomed out view of the docking of acetate compounds in candidate enzymes

Docking of Z7-16:OAc (display in violet), Z9-16:OAc (in turquoise green) and Z11-16:OAc (in orange) into the predicted 3D structure of *H. subflexa* (**a**, **c**, **e**, **g**) and *H. virescens* (**b**, **d**, **f**, **h**) allele of LipX (**a**, **b**), LipZ (**c**, **d**), Est1 (**e**,**f**) and Est2 (**g**, **h**). Residues that are expected to belong to the catalytic triads based on homology are shown as ball and sticks and colored in magenta. Displayed oxygen, hydrogen, nitrogen and sulfur atoms are respectively colored in red, white, blue and yellow, respectively. Predicted hydrogen bonds are displayed as dashed cyan lines.

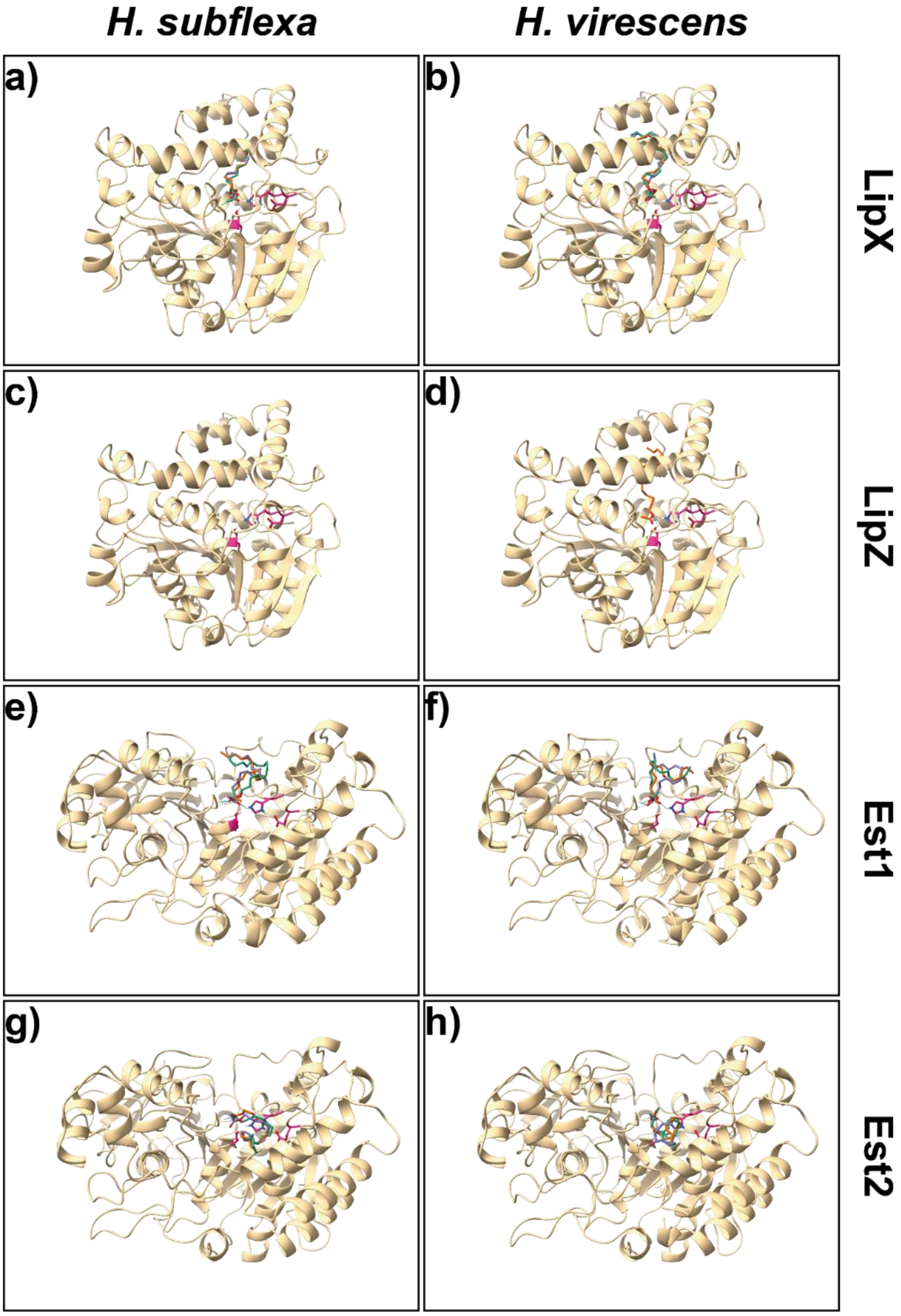

Supplementary Fig. S5. CRISPR/Cas9-induced mutations in the *H. virescens* allele of LipX.

Screening primers (VVLipX-scr-Fd and reverse-complement of VVLipX-scr-R are shown) produce a 265 nt amplicon from the wild-type (wt) gene, with amino acid sequence indicated below (catalytic serine in red). Two guide RNAs with PAM (in bold) are shown (VVLipX-T1 and reverse-complement of VVLipX-T2). Allele -5GGGAA has one deletion of 5 nt producing a frameshift; the predicted protein sequence is shown in Supplementary Fig. S10. Allele -5ATTCC also has one deletion of 5 nt producing the same frameshift.

```
VVLipX-scr-Fd-->
CGATGATCGACYATGTTCTGGA
CGATGATCGACTATGTTCTGGACTACACAGGATTCGAGAAGCTGAACT wt
CGATGATCGACTATGTTCTGGACTACACAGGATTCGAGAAGCTGAACT -5GGGAA
CGATGATCGACTATGTTCTGGACTACACAGGATTCGAGAAGCTGAACT -5ATTCC
   M  I  D  Y  V  L  D  Y  T  G  F  E  K  L  N

            VVLipX-T1-->
             CCAAGGAACGGGAACATTCCTGG
ACATTGGTTTTTCCCAAGGAACGGGAACATTCCTGGTGATGTGCTCCG wt
ACATTGGTTTTTCCCAAGGAAC:::::CATTCCTGGTGATGTGCTCCG -5GGGAA
ACATTGGTTTTTCCCAAGGAACGGGAAC:::::TGGTGATGTGCTCTG -5ATTCC
Y  I  G  F  S  Q  G  T  G  T  F  L  V  M  C  S

AGAGGCCGGGATACTGTGATAAAGTGAAATTAGTGATTACTTTAGCGC wt
AGAGGCCGGGATACTGTGATAAAGTGAAATTAGTGATTACTTTAGCGC -5GGGAA
AGAGGCCGGGATACTGTGATAAAGTGAAATTAGTGATTACTTTAGCGC -5ATTCC
E  R  P  G  Y  C  D  K  V  K  L  V  I  T  L  A

                            <--VVLipX-T2
                         CCAGGATATTTAGGACTATGA
CTGCAAGCAGGCAGATGCATACACAGTCCAGGATATTTAGGACTATGA wt
CTGCAAGCAGGCAGATGCATACACAGTCCAGGATATTTAGGACTATGA -5GGGAA
CTGCAAGCAGGCAGATGCATACACAGTCCAGGATATTTAGGACTATGA -5ATTCC
P  A  S  R  Q  M  H  T  Q  S  R  I  F  R  T  M

CT
CCCAAACGTTTTATAGAATGGAAAGCGTGTTATCAATGACAGGTTTAC wt
CCCAAACGTTTTATAGAATGGAAAGCGTGTTATCAATGACAGGTTTAC -5GGGAA
CCCAAACGTTTTATAGAATGGAAAGCGTGTTATCAATGACAGGTTTAC -5ATTCC
T  Q  T  F  Y  R  M  E  S  V  L  S  M  T  G  L

     <--VVLipX-scr-R
     GTGTTTCCTAAAGGCGGCTT
AAGAAGTGTTTCCTAAAGGCGGCTT wt
AAGAAGTGTTTCCTAAAGGCGGCTT -5GGGAA
AAGAAGTGTTTCCTAAAGGCGGCTT -5ATTCC
Q  E  V  F  P  K  G  G
```

Supplementary Fig. S6. CRISPR/Cas9-induced mutations in the *H. virescens* allele of LipZ.

Screening primers (VVLipZ-scr-F and reverse-complement of VVLipZ-scr-R are shown) produce a 317 nt amplicon from the wild-type (wt) gene, with amino acid sequence indicated below (catalytic serine in red). Two guide RNAs with PAM (in bold) are shown (VVLipZ-T1 and reverse-complement of VVLipZ-T2). Allele -13-1 has one deletion of 13 nt and one deletion of 1 nt producing a frameshift; the predicted protein sequence is shown in Supplementary Fig. S10. Allele -21-12 has one deletion of 21 nt and another deletion of 12 nt. This does not cause a frameshift, but the first deletion removes the serine of the catalytic triad; therefore, the predicted protein sequence shown in Supplementary Fig. S10 has no lipase enzymatic activity.

```
VVLipZ-scr-F-->
GATGGGAGTCTTCGATGTGC
GATGGGAGTCTTCGATGTGCCCGCGATGATAGACCATGTTCTAAATTA wt
GATGGGAGTCTTCGATGTGCCCGCGATGATAGACCATGTTCTAAATTA -13-1
GATGGGAGTCTTCGATGTGCCCGCGATGATAGACCATGTTCTAAATTA -21-12
 M  G  V  F  D  V  P  A  M  I  D  H  V  L  N  Y

                                       VVLipZ-T1-->
                                       TCAAGGCACTGG
TACGAGATCTGAGAAGCTCAACTACATGGGTTTTTCTCAGGGTACTGG wt
TACGAGATCTGAGAAGCTGAACTACATCGGTTTTTCTCA::::::::: -13-1
TACGAGATCTGAGAAGCTGAACTACATCGAATTT:::::::::::::: -21-12
 T  R  S  E  K  L  N  Y  M  G  F  S  Q  G  T  G

GACATTTCTGG
GACATTCCTGGTGATGTGTTCCGAGAAACCAGGATATTGTGACAAAGT wt
::::TT:CTGGTGATGTGTTCCGAAAAACCAGGATATTGTGATAAAGT -13-1
::::::CTGGTGATGTGTTCCGAAAAACCAGGATATTGTGATAAAGT -21-12
 T  F  L  V  M  C  S  E  K  P  G  Y  C  D  K  V

                          <--VVLipZ-T2
                        CCAGCAAGTAGACATAAAAATTC
CAATTTACTGATAGCTTTATCACCAGCGAGTAGGCATAAAAATTCACA wt
GAATTTACTGATAGCTTTATCACCAGCAAGTAGACATAAAAATTCACA -13-1
GAATTTACTGATAGCTTTATCACCAGC::::::::::::AAATTCACA -21-12
 N  L  L  I  A  L  S  P  A  S  R  H  K  N  S  Q

GTCGAGGATATTCAGATCTGTGACGCAGATGTTTGAACGACTGGAAGA wt
GTCGAGGATATTCAGATCTGTGACGCAGATGTTTGAACGACTGGAAGA -13-1
GTCGAGGATATTCAGATCTGTGACGCAGATGTTTGAACGACTGGAAGA -21-12
 S  R  I  F  R  S  V  T  Q  M  F  E  R  L  E  D

CATGTTATCAATGGCTGGTTTATACGAAGCACTTTCTAAAGGTGGACC wt
CATGTTATCAATGGCTGGTTTATACGAAGCACTTTCTAAAGGTGGACC -13-1
CATGTTATCAATGGCTGGTTTATACGAAGCACTTTCTAAAGGTGGACC -21-12
 M  L  S  M  A  G  L  Y  E  A  L  S  K  G  G  P

           <--VVLipZ-scr-R
          TTTGGTGCATTCTTCTGCC
CAGTCAGGAGTTTGGTGCTTTCTTCTGCC wt
CAGTCAGGAGTTTGGTGCATTCTTCTGCC -13-1
CAGTCAGGAGTTTGGTGCATTCTTCTGCC -21-12
 S  Q  E  F  G  A  F  F  C
```

Supplementary Fig. S7. CRISPR/Cas9-induced mutations in the *H. virescens* allele of Est1.

Screening primers (VVest1-scr-Fd and reverse-complement of VVest-scr-R are shown) produce a 328 nt amplicon from the wild-type (wt) gene, with amino acid sequence indicated below (catalytic serine in red). Two guide RNAs with PAM (in bold) are shown (VVest1-T2 and reverse-complement of VVest1-T1). Allele -2 has one deletion of 2 nt producing a frameshift; allele +5 has an insertion of 5 nt also producing a frameshift. Predicted protein sequences are shown in Supplementary Fig. S11.

```
VVest1-scr-Fd-->
CCTGCAGTGTCTCMGACTCAA
CCTGCAGTGTCTCAGACTCAACTTATATGTGCCACACACGGCCAGCCC wt
CCTGCAGTGTCTCAGACTCAACTTATATGTGCCACACACGGCCAGCCC -2
CCTGCAGTGTCTCAGACTCAACTTATATGTGCCACACACGGCCAGCCC +5
  L  Q  C  L  R  L  N  L  Y  V  P  H  T  A  S  P

CAAGAACCTATTGCCAATATTGGTCTGGTTCCACGGCGGAGGATTTGC wt
CAAGAACCTATTGCCAATATTGGTCTGGTTCCACGGCGGAGGATTTGC -2
CAAGAACCTATTGCCAATATTGGTCTGGTTCCACGGCGGAGGATTTGC +5
  K  N  L  L  P  I  L  V  W  F  H  G  G  G  F  A

                                    <--VVest1-T1
                           CCAATA:::::TTTGGTCAAA
TTTCGGCAGCGCTGGCGAATATGGCGGCCAATA:::::TTTGGTCAAA wt
TTTCGGCAGCGCTGGCGAATATGGCGGCC::TA:::::TTTGGTCAAA -2
TTTCGGCAGCGCTGGCGAATATGGCGGCCAATAATTATTTTGGTCAAA +5

  F  F  S  A  G  E  Y  G  G  Q  Y        L  V  K

CAAGACA
CAAGACATCATCGTTGTCACAGTTAACTACAGACAAGGGGCATATGGC wt
CAAGACATCATCGTTGTCACAGTTAACTACAGACAAGGGGCATATGGC -2
CAAGACATCATCGTTGTCACAGTTAACTACAGACAAGGGGCATATGGC +5
 Q  D  I  I  V  V  T  V  N  Y  R  Q  G  A  Y  G

                       VVest1-T2-->
                  CGGAATGTCACTGGCAATCAGGG
TTCTTGTGTTTGAACGATCGGAATGTCACTGGCAACCAGGGTATGAAA wt
TTCTTGTGTTTGAACGATCGGAATGTCACTGGCAACCAGGGTATGAAA -2
TTCTTGTGTTTGAACGATCGGAATGTCACTGGCAACCAGGGTATGAAA +5
 F  L  C  L  N  D  R  N  V  T  G  N  Q  G  M  K

GACCAGATCGAAGCTCTAAGGTGGATCAAAAGACACATAGCTAATTTT wt
GACCAGATCGAAGCTCTAAGGTGGATCAAAAGACACATAGCTAATTTT -2
GACCAGATCGAAGCTCTAAGGTGGATCAAAAGACACATAGCTAATTTT +5
 D  Q  I  E  A  L  R  W  I  K  R  H  I  A  N  F

                          <--VVest1-scr-R
                          TGCTGGTGAAAGCTATGGC
AGTGGTGATCCTAGCAAAGTCACTATTGCTGGTGAAAGCTATGGC wt
AGTGGTGATCCTAGCAAAGTCACTATTGCTGGTGAAAGCTATGGC -2
AGTGGTGATCCTAGCAAAGTCACTATTGCTGGTGAAAGCTATGGC +5
 S  G  D  P  S  K  V  T  I  A  G  E  S  Y  G
```

Supplementary Fig. S8. CRISPR/Cas9-induced mutations in the *H. virescens* allele of Est2.

Screening primers (VVest2-scr-F and reverse-complement of VVest2-scr-R are shown) produce a 318 nt amplicon from the wild-type (wt) gene, with amino acid sequence indicated below (catalytic serine in red). Two guide RNAs with PAM (in bold) are shown (VVest2-T2 and reverse-complement of VVest2-T1). Allele -1 has one deletion of 1 nt producing a frameshift; allele +5+24 has an insertion of 5 nt followed by another insertion of 24 nt, also producing a frameshift. Predicted protein sequences are shown in Supplementary Fig. S11.

```
VVest2-scr-F-->
CCACATACAGCCAACGAGAA
CCACATACAGCCAACGAGAACAACACAGTGCCAATATTGGTGTACTTC wt
CCACATACAGCCAACGAGAACAACACAGTGCCAATATTGGTGTACTTC -1
CCACATACAGCCAACGAGAACAACACAGTGCCAATATTGGTGTACTTC +5+24
 P  H  T  A  N  E  N  N  T  V  P  I  L  V  Y  F


                                              CCAA
TACGGAGGTGGCTTCATGTTCGGCAGCGCCAGTGAGTATGGTGGCCAA wt
TACGGAGGTGGCTTCATGTTCGGCAGCGCCAGTGAGTATGGTGGCCAA -1
TACGGAGGTGGCTTCATGTTCGGCAGCGCCAGTGAGTATGGTGGCCAA +5+24
 Y  G  G  G  F  M  F  G  S  A  S  E  Y  G  G  Q


       <--VVest2-T1
CA::::TTTGGTCAAACACGACA
CA::::TTTGGTCAAACACGACATCATTGTCATCACAGTGAACTACA wt
C:::::TTTGGTCAAACACGACATCATTGTCATCACAGTGAACTACA -1
CAAACGGTTTGGTCAAACACGACATCATTGTCATCACAGTGAACTACA +5+24
 H        L  V  K  H  D  I  I  V  I  T  V  N  Y


GACTTGGTCCTTACGGCTTCTTGTGCTTGAACGATGAAACTGTACCTG wt
GACTTGGTCCTTACGGCTTCTTGTGCTTGAACGATGAAACTGTACCTG -1
GACTTGGTCCTTACGGCTTCTTGTGCTTGAACGATGAAACTGTACCTG +5+24
R  L  G  P  Y  G  F  L  C  L  N  D  E  T  V  P


                                      VVest2-T1-->
                                       ATGGATAAGGA
GCAACCAAGGGCTTAAAGACCAGATAGCCGCGTTGAGATGGATAAGGA wt
GCAACCAAGGGCTTAAAGACCAGATAGCCGCGTTGAGATGGATAAGGA -1
GCAACCAAGGGCTTAAAGACCAGATAGCCGCGTTGAGATGGATAAGGA +5+24
G  N  Q  G  L  K  D  Q  I  A  A  L  R  W  I  R


AAAAT:::::::::::::::::::::::ATAGAGG
AAAAT:::::::::::::::::::::::ATAGAGGCTTTTGGCGGAG wt
AAAAT:::::::::::::::::::::::ATAGAGGCTTTTGGCGGAG -1
AAAATTAGCTTTTCTTGAATCTTAATAGAATAGAGGCTTTTGGCGGAG +5+24
K  N                          I  E  A  F  G  G


                                   <--VVest2-scr-R
                                         GAGGTGGCG
ATCCTTATAAAGTGACCATTTCTGGCCAAAGCTATGGAGGAGGTGGCG wt
```

ATCCTTATATAAAGTGACCATTTCTGGCCAAAGCTATGGAGGAGGTGGCG -1
ATCCTTATATAAAGTGACCATTTCTGGCCAAAGCTATGGAGGAGGTGGCG +5+24
D P Y K V T I S G Q S Y G G G G

<--VVest2-scr-R
TAGATTTGCAT
TAGATTTGCAT wt
TAGATTTGCAT -1
TAGATTTGCAT +5+24
V D L H

Supplementary Fig. S9: CRISPR/Cas9 experimental scheme

Scheme of the injections and crossings performed for the CRISPR/Cas9 LipX, LipY, Est1 and Est2 gene inactivation.

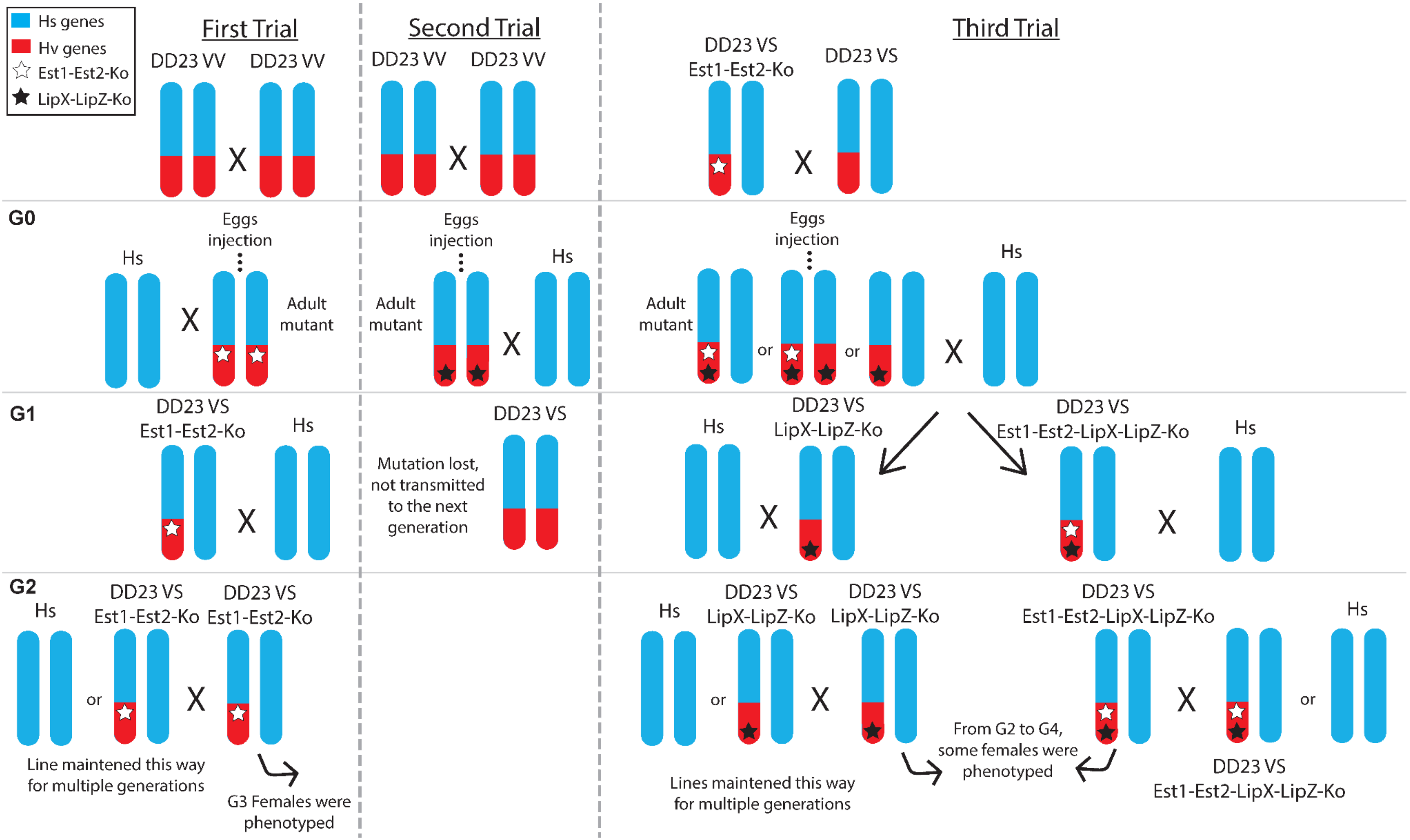

Supplementary Fig. S10. Alignment of LipX and LipZ protein sequences used in the modelling and docking studies.

The putative signal peptides have been removed. Residues of the catalytic triad are in red. Canine Gastric Lipase (pdb|1K8Q|A) is shown for reference. Predicted protein sequences of CRISPR/Cas9-generated mutants are also shown. All are truncated by premature stop codons except for HvirLipZ-21-12, which has an in-frame deletion including the catalytic serine.

```
pdb|1K8Q|A         ------------AFGKLHPTNPEVTMNISQMITYWGYPAEEYEVVTEDGYILGIDRIPYG
HvirLipX           ----SPSLYLRRETKSSLGYPKDSLLNFTELTAEYGYVSEEHKVVTEDGYILTMFRIVKA
HvirLipX-5-GGGAA   ----SPSLYLRRETKSSLGYPKDSLLNFTELTAEYGYVSEEHKVVTEDGYILTMFRIVKA
HvirLipX-5-ATTCC   ----SPSLYLRRETKSSLGYPKDSLLNFTELTAEYGYVSEEHKVVTEDGYILTMFRIVKA
HsubLipX           ----SPSLYLRRETKSSLGYPKDSLLNFTELTAEYGYVSEEHKVVTEDGYILTMFRIVKA
HvirLipZ           QVLRSPSLHLRREAKSSLGYPKDSLLNFTELTAEYGYVSEEHKVVTEDGYILTMFRIVKA
HvirLipZ-13-1      QVLRSPSLHLRREAKSSLGYPKDSLLNFTELTAEYGYVSEEHKVVTEDGYILTMFRIVKA
HvirLipZ-21-12     QVLRSPSLHLRREAKSSLGYPKDSLLNFTELTAEYGYVSEEHKVVTEDGYILTMFRIVKA
HsubLipZ           QVLRSPNLHLRQETKSSLGYTKDSLLNFTELTAEYGYVSEEHKVVTDDGYILTMFRIAKA


pdb|1K8Q|A         RKNSENIGRRPVAFLQHGLLASATNWISNLPNNSLAFILADAGYDVWLGNSRGNTWARRN
HvirLipX           R-NCHRAKRSPPVLLMHGLLQSSDSWIDSGPNAGLAYLISDACYDLWLGNVRGNYYSRGH
HvirLipX-5-GGGAA   R-NCHRAKRSPPVLLMHGLLQSSDSWIDSGPNAGLAYLISDACYDLWLGNVRGNYYSRGH
HvirLipX-5-ATTCC   R-NCHRAKRSPPVLLMHGLLQSSDSWIDSGPNAGLAYLISDACYDLWLGNVRGNYYSRGH
HsubLipX           R-NCHRAKRSPPVLLMHGLLQSSDSWIDSGPNAGLAYLISDACYDLWLGNVRGNYYSRGH
HvirLipZ           R-NCHRAKRSPPVLLMHGLLQSSDSWIDSGPNAGLAYLISDACYDLWLGNVRGNYYSRGH
HvirLipZ-13-1      R-NCHRAKRSPPVLLMHGLLQSSDSWIDSGPNAGLAYLISDACYDLWLGNVRGNYYSRGH
HvirLipZ-21-12     R-NCHRAKRSPPVLLMHGLLQSSDSWIDSGPNAGLAYLISDACYDLWLGNVRGNYYSRGH
HsubLipZ           R-NCHRAKRSPPVLLMHGLLQSSDSWIDSGPNAGLAYLISDACYDLWLGNVRGNYYSRGH


pdb|1K8Q|A         LYYSPD-SVEFWAFSFDEMAKYDLPATIDFILKKTGQDKLHYVGHSQGTTIGFIAFSTNP
HvirLipX           VRLDPDKDPAYWKFYIEQIGIYDVPAMIDYVLDYTGFEKLNYIGFSQGTGTFLVMCSERP
HvirLipX-5-GGGAA   VRLDPDKDPAYWKFYIEQIGIYDVPAMIDYVLDYTGFEKLNYIGFSQGTIPGDVLREAGI
HvirLipX-5-ATTCC   VRLDPDKDPAYWKFYIEQIGIYDVPAMIDYVLDYTGFEKLNYIGFSQGTIPGDVLREAGI
HsubLipX           VRLDPDKDPAYWKFYIEQIGIYDVPAMIDYVLDYTGFEKLNYIGFSQGTGTFLVMCSERP
HvirLipZ           VRLNPDKDPAYWKFYIDEMGVFDVPAMIDHVLNYTRSEKLNYIGFSQGTGTFLVMCSEKP
HvirLipZ-13-1      VRLDPDKDPAYWKFYIDEMGVFDVPAMIDHVLNYTRSEKLNYMGFSHSGDVFRETRIL--
HvirLipZ-21-12     VRLDPDKDPAYWKFYIDEMGVFDVPAMIDHVLNYTRSEKLNYI-------EFLVMCSEKP
HsubLipZ           VRLDPDKDPAYWKFYIEQMGVYDVPVMIDYVLKHTRSEKLNYIGFSQGTGTFLVMCSEKP
```

```
pdb|1K8Q|A         KLAKRIKTFYALAPVATVKYTETLINKLMLVPSFLFKLIFG----NKIFYPHHFFDQFLA
HvirLipX           GYCDKVKLVITLAPASRQMHTQSRIFRTMTQTFYRMESVLSMTGLQEVFPKGGFSQEFVA
HvirLipX-5-GGGAA   L-----------------------------------------------------------
HvirLipX-5-ATTCC   L-----------------------------------------------------------
HsubLipZ           GYCEKVNFLIALSPASRHKNSQSKIFRSVTQMFEQLEDMLSMAGLYEALSKGGPSQEFGA
HvirLipZ           GYCDKVNLLIALSPASRHKNSQSRIFRSVTQMFERLEDMLSMAGLYEALSKGGPSQEFGA
HvirLipZ-21-12     GYCDKVNLLIALSPA----NSQSRIFRSVTQMFERLEDMLSMAGLYEALSKGGPSQEFGA
HsubLipX           GYCDKVKLVITLAPASRQMHTQSRIFRTMTQTFYRMESVLSMTGLQEVFPKGGFSQEFVA

pdb|1K8Q|A         TEVCSRETVDLLCSNALFIICGFDTMNLNM---SRLDVYLSHNPAGTSVQNVLHWSQAVK
HvirLipX           FFCQLSGVTERLCERVIDVFDHVDTTHLGSITNQTTRVLFGHFPAGTSVHNMARYGQSMN
HsubLipX           FFCQLSGVTERLCEKVIDVFDHVDSTHLGSITNQTTRVLFGHFPAGTSVHNMARYGQSMN
HvirLipZ           FFCHLSGETVKMCEEAIYEFDHVNSLHFGSITNKTRRALFGHFPAGTSVHNMARYGQSMR
HvirLipZ-21-12     FFCHLSGETVKMCEEAIYEFDHVNSLHFGSITNKTRRALFGHFPAGTSVHNMARYGQSMR
HsubLipZ           FFCHLSGETEKMCEEAIYEFDHVNSLHFGSITNKTRRVLFGHFPAGTSVHNMARYGQSMR

pdb|1K8Q|A         SGKFQAFDWGSPVQNMMHYHQSMPPYYNLTDMHVPIAVWNGGNDLLADPHDVDLLLSKLP
HvirLipX           SGRFEKFNYGKE-QNLVVYGSEEPPQYNLSATTVPVMCIYGKNDGLVDTRDVEWLMSQLP
HsubLipX           SGRFEKFNYGKE-QNLVLYGSEEPPQYNLSATTVPVLCIYGKNDGLVDTRDVEWLMSQLP
HvirLipZ           SRRFEKFNYGKE-QNLVLYGSEEPPQYNLSATTVPVMCIYGKNDGVVDLRDVEWLMSQLP
HvirLipZ-21-12     SRRFEKFNYGKE-QNLVLYGSEEPLQYNLSATTVPVMCIYGKNDGVVDLRDVKWLMSQLP
HsubLipZ           SRRFEKFNYGKE-QNLVLYGSEEPPQYNLSATTVPVLCIYGKNDGVVDLRDVEWLMSQLP

pdb|1K8Q|A         NLIYHRKI--PPYNHLDFIWAMDAPQAVYNEIVSMM---GTD
HvirLipX           NVLEMVKVEDPQWNHMDVTYSQYTGDTIFPKINEYLLKYTSA
HsubLipX           NVLEMVKVEDPQWNHMDVTYSQYTGDTIFPKINEYLLKYTSA
HvirLipZ           NVLEMMKVEDPQWSHLDVTYSQYTKDIIFPKIYEYLLKFSHS
HvirLipZ-21-12     NVLEMMKVEDPQWSHLDVTYSQYTKDIIFPKIYEYLLKFSHS
HsubLipZ           NVLEMMKVEDPQWSHLDVTYSQYTKDTIFPKIYEYLLKFSHS
```

Supplementary Fig. S11. Alignment of Est1 and Est2 protein sequences used in the modelling and docking studies.

The putative signal peptides have been removed. Residues of the catalytic triad are in red. *Epiphyas postvittana* antennal carboxylesterase-24 (pdb|7MP4|A) is shown for reference. Predicted protein sequences of CRISPR/Cas9-generated mutants are also shown. All are truncated by premature stop codons prior to the catalytic triad.

```
pdb|7MP4|A       -----SKPVVRVTQGVLQGSWKVSTHGRTYASFEGVPYAR----PPVGKYRFREPQHLKP
HvirEst1         ----EDTVTVQTNKGAIQGTIKDG-----YNTFFGIPYAKVNEENPFGRTLDY-PKFNTP
HvirEst1-2       ----EDTVTVQTNKGAIQGTIKDG-----YNTFFGIPYAKVNEENPFGRTLDY-PKFNTP
HvirEst1+5       ----EDTVTVQTNKGAIQGTIKDG-----YNTFFGIPYAKVNEENPFGRTLDY-PKFNTP
HsubEst1         EGTDNKTVIVETSKGAIQGNIKDG-----YNTFFGIPYAKVNEQNPFGRTLDY-PKFKTP
HvirEst2         ----QENVTVMTSKGPIVGFKQDG-----YNTFLGIPYALVNEVNPFGNNLVY-PDFQTP
HvirEst2-1       ----QENVTVMTSKGPIVGFKQDG-----YNTFLGIPYALVNEVNPFGNNLVY-PDFQTP
HvirEst2+5+24    ----QENVTVMTSKGPIVGFKQDG-----YNTFLGIPYALVNEVNPFGNNLVY-PDFQTP
HsubEst2         ----EENVTVMTSKGPIVGFKQDG-----YNTFLGVPYALVNEDNPFGNNLVH-PDFKTP

pdb|7MP4|A       WAGVWDASKTLPQCLQWDPFQQEVSGSENCLYINVHTP-KLSAGASLPVVVFIHGGAFMY
HvirEst1         YIAN-DSSVICPQVY------FNDNGVLQCLRLNLYVPHTASPKNLLPILVWFHGGGFAF
HvirEst1-2       YIAN-DSSVICPQVY------FNDNGVLQCLRLNLYVPHTASPKNLLPILVWFHGGGFAF
HvirEst1+5       YIAN-DSSVICPQVY------FNDNGVLQCLRLNLYVPHTASPKNLLPILVWFHGGGFAF
HsubEst1         YIAN-DSSVICPQVY------FNDKGVLQCLRLNIYVPHKASAKNLLPILVWFHGGGFAF
HvirEst2         FMAN-DGSIICPQVI------TRVGGVLQCLRMNMYVPHTANENNTVPILVYFYGGGFMF
HvirEst2-1       FMAN-DGSIICPQVI------TRVGGVLQCLRMNMYVPHTANENNTVPILVYFYGGGFMF
HvirEst2+5+24    FMAN-DGSIICPQVI------TRVGGVLQCLRMNMYVPHTANENNTVPILVYFYGGGFMF
HsubEst2         FIAN-DGSIICPQVI------SRVGGVLQCLRMNIYVPHTANENNTVPILVYFYGGGFMF

pdb|7MP4|A       GAGSLYDVSHLMDRDVVAVTFNYRLGPLGFLSTGDESAPGNAGLKDQAFALQWVKNNVMM
HvirEst1         GSAGEYGGQYLVKQDIIVVTVNYRQGAYGFLCLNDRNVTGNQGMKDQIEALRWIKRHIAN
HvirEst1-2       GSAGEYGGLFGQTRHYRCHSELQTRGLWLLVFERSECHWQSGYERPDRSFKVDQKTYS--
HvirEst1+5       GSAGEYGGQ---------------------------------------------------
HsubEst1         GSAGEYGGQYLVKQDIIVITVNYRQGAYGFLCLNDRNVTGNQGMKDQIEALRWIKKHIAN
HvirEst2         GSASEYGGQHLVKHDIIVITVNYRLGPYGFLCLNDETVPGNQGLKDQIAALRWIRKNIEA
HvirEst2-1       GSASEYGGQ--LWSNTTSLSSQ--------------------------------------
HvirEst2+5+24    GSASEYGGQQTVWSNTTSLSSQ--------------------------------------
HsubEst2         GSASEYGGQHLVKHDIIVITVNYRLGPYGFLCLNDESVPGNQGLKDQIAALRWIRKNIDA
```

```
pdb|7MP4|A        FGGNPDSVTLTGCSAGGASVHYHYLSPLSKGNFARGIAFSGAAFASWTHAVKPLQNAR-S
HvirEst1          FSGDPSKVTIAGESYGGGAVDLHLYSKY-ETLFQKAIVQSGSIFVTEGIFIKPDYDAAIK
HsubEst1          FSGDPKKVTIAGESYGGGGVDLHLYSKY-ETLFQKAIVQSGSIFVTEGIFIKPDYDAAIK
HvirEst2          FGGDPYKVTISGQSYGGGGVDLHLYSKY-ETLFDKAIIQSGSIY-TPGFYGNREHDAAIK
HsubEst2          FGGDPYKVTISGQSYGGGSVDLHLYSKY-ETLFDKAIIQSGSIY-TPGFYGNREHDAAIK

pdb|7MP4|A        LAAIVGCPTGTNRELVDCLKYRPAEVVVGAQIEMLEFPYQQMFTPFTPTVEPQGTRDAFL
HvirEst1          LAKYLGHNVTTTPNALKVLAKAKPADVNAATRNL------SMILTLCKEKKFKGIPN-FA
HsubEst1          LAKYLGHNVTTTSKALKVLAKAKPVDVNAATRNL------SMILTLCKEKKFKGIPN-FV
HvirEst2          IALHLGYRVMSTQEALELLAKENPLVVMAAARNL------SMRLTACKEIRFKGIQN-FV
HsubEst2          IALHLGYRVLSTQEALEFLAKENPLVVMAAARNL------SMRLTACKEIRFKGIQN-FV

pdb|7MP4|A        TQYPFLVAQAGGMHKVPLITSVTSEEGLYPAAVYQKSPDTLAYLEANWDQLASNIFEYND
HvirEst1          TDDPFHLNKPERINRTSIMIGYTSQEMLYEFVNKPQS------VYDKLGNPFLPQLSKTF
HsubEst1          TDDPFHLNKPERINRTSIMIGYTSQEMLYEFVNKSQS------VYDKLGNPFLPQLSKTF
HvirEst2          TKCLNHLHNPDRIENIPIMIGYNSKEDFGTFANKPQQ------FYDSLGDIFYRNLQINF
HsubEst2          TKCLNHLHNPDRIENIPIMIGYNSKEDFGTFANKPQQ------FYDSLGDIFYRNLQINF

pdb|7MP4|A        TLPVNQRAGVAAKIKQRYLGNKPVSQETYPQLVQALGDRLFAVDVGKLAQIHARHSGQPT
HvirEst1          ALPKNELERLSNIIQHFYLGGKKIGPDSQLELSNFLSDFAINYGAEWSVNRYMAQKAKAV
HsubEst1          ALPKNELERLSNIIQHFYLGGKAIGPDTQLELSNFLSDFAINYGAEWSVNRYIAQRAKAV
HvirEst2          VLKTCEMETLSNITRRFYMGSKNIGPESMLELTDYSSDFKLNYAVEKSVSKYIEQGGK-V
HsubEst2          VLKTCEMETLSNITRTFYMGSKNIGPESILELTDYSSDFKLNYAVEKSVSKYIEQGGK-V

pdb|7MP4|A        YLYRYSFRGEKSLSNMMASNDKNYGVSHADDIFHIFKFPSLS-STSSEDVRMTEALIDMI
HvirEst1          YKYVFSYIGGSEYKNVTGA-----GASHTEELKYLFNWVWANPPSTPEQVLIRDRMTTMW
HsubEst1          YKYVFSYIGGSDYQNVTGA-----GASHTEELKYLFNWVWANPPSTPEQVLIRDRMTTMW
HvirEst2          YKYMFSYIGGSQYKNVTGA-----GATHTEELKYLFETTWK--LTSDEQRMMRDRMTTMW
HsubEst2          YKYMFSYIGGSQYKNVTGA-----GATHTEELKYLFETSWK--LTSDEQRMMRDRMTTMW

pdb|7MP4|A        YSFSTTGNPKLTNEAPV---WTPVTPGSAELSYLEIASPSRMEMKSSSDFGHRSFWDSLG
HvirEst1          ANFVKFGNPTPTVTELLPVTWTPVSGSARPYLNIDTN----MQMKDFTFKHRLAFWELFW
HsubEst1          ANFVKFGNPTPKETELLPVTWTPVTGSARPYLNIDTD----MQMRDFAFKHRLAFWELFW
HvirEst2          ANFVKLGNPTPQKTDLLPVTWNPVNGKARPYLDIDVT----MSMKEHAYRHRIAFWELFM
HsubEst2          ANFVKLGNPTPQKTDLLQVTWDPVNSKARPYLDIDVT----MSMKEHAYRHRMAFWELFM

pdb|7MP4|A        FVENENYRHIRDELENLYFQG
```

```
HvirEst1         HMYGKKHVLLSERKKKNKNKN
HsubEst1         HMYGKKHVLLSERKKKNKNKN
HvirEst2         LKYDKRS--------------
HsubEst2         LKYDKRS--------------
```

Supplementary Table S1: DD23 female pheromone gland unfiltered and reference transcriptomes descriptive metrics.

Table displaying descriptive metrics of the unfiltered and the reference transcriptomes of DD23 female pheromone glands. Lowcovered contig number, segmented contig number and assembly score were obtained from Transrate[61]. One-copy BUSCO, duplicated BUSCO and missing BUSCO numbers were obtained from BUSCO[60].

Transcriptome	Contig number	N50	GC percent	Lowcovered contigs	Segmented contigs	Assembly Score	One-copy Busco	Duplicated Busco	Missing Busco
Unfiltered	93659	2571	40,31%	49096	14154	0,247	949	562	38
Reference	18343	2603	42,04%	0	3560	0,317	1247	233	78

Supplementary Table S2: Differential expression statistics and annotation of differentially expressed contigs in the DD23 pheromones glands reference transcriptome.

Table displaying base 2 log fold-change in expression (LogFC) and associated adjusted *p* value (Adj pvalue) obtained from DESeq2 (see Love *et al.* 2014), false discovery rate (FDR) from edgeR (see Robinson *et al.* 2009)as well as Blast2GO annotation and best blastx hit for differentially expressed contig in the DD23 female pheromone gland reference transcriptome.

Transcript ID	Log2FC	Adj pvalue	FDR	Blast2GO Description	Besthit NR ID
comp12227_c0_seq1	2,52	2,33E-09	1,99E-07		
comp24491_c0_seq3	-3,04	8,97E-15	3,93E-09	Pacifastin-like protease inhibitor cvp4	XP_013137917.1
comp27469_c0_seq2	-2,05	6,05E-07	1,94E-04	hypothetical protein KGM_06068	EHJ64284.1
comp28829_c0_seq1	-4,06	7,03E-26	6,48E-33	myrosinase 1-like	XP_012545806.1
comp30551_c0_seq2	-2,33	6,42E-08	1,60E-06	endonuclease-reverse transcriptase	XP_011555154.1
comp31652_c0_seq3	-3,61	3,07E-20	1,08E-22	myrosinase 1-like	KPJ04243.1
comp32219_c0_seq1	2,90	3,65E-22	1,89E-07	SOSS complex subunit B homolog	XP_004927331.1
comp32945_c0_seq1	-2,35	4,53E-08	7,17E-07		
comp34660_c3_seq2	-3,90	5,75E-35	7,21E-13		
comp34863_c0_seq1	-3,50	1,68E-20	1,06E-11	kxDL motif-containing CG10681	NP_001299090.1
comp34863_c0_seq2	3,48	5,62E-32	4,55E-11	kxDL motif-containing CG10681	NP_001299090.1
comp35374_c0_seq1	3,64	3,29E-21	1,10E-14	tetratricopeptide repeat 4	XP_004922588.1
comp35374_c0_seq2	2,21	4,16E-21	8,80E-07	tetratricopeptide repeat 4	XP_004922588.1
comp35589_c0_seq1	3,87	8,33E-26	2,96E-13	probable ATP-dependent RNA helicase DDX27	XP_012545812.1
comp35589_c0_seq2	-2,95	1,50E-13	2,09E-09	probable ATP-dependent RNA helicase DDX27	XP_012545812.1
comp35597_c0_seq2	-3,16	4,93E-16	1,41E-09	GTP-binding RAD	XP_013165551.1
comp35971_c1_seq1	-2,28	1,76E-07	1,48E-06	f-box lrr-repeat 7	XP_013137848.1
comp36091_c0_seq1	-3,88	8,08E-27	3,41E-13	snRNA-activating complex subunit 1	XP_013137921.1
comp36091_c0_seq2	4,83	3,94E-65	8,68E-21	snRNA-activating complex subunit 1	XP_013137921.1
comp36176_c0_seq3	2,46	8,74E-22	1,08E-05	Diguanylate cyclase with PAS sensor	XP_013191729.1
comp36300_c0_seq1	-2,02	7,46E-08	1,24E-04	NACHT domain- and WD repeat-containing 1	XP_013191595.1

comp36335_c1_seq1	4,10	8,20E-30	1,32E-14	DNA ligase 1	JAT88061.1
comp36335_c1_seq3	-3,52	9,19E-21	9,19E-12	DNA ligase 1	JAT88061.1
comp36643_c0_seq11	2,46	7,38E-09	3,93E-09	THAP domain-containing 1-like	WP_047149324.1
comp36702_c0_seq1	6,46	1,28E-101	1,03E-40	SDE2 homolog	XP_013165341.1
comp36702_c0_seq2	-5,88	2,01E-78	1,16E-35	SDE2 homolog	XP_013165341.1
comp36915_c0_seq1	-3,67	3,26E-23	2,73E-12	Origin recognition complex subunit 5	KOB72064.1
comp36915_c0_seq2	3,77	1,46E-24	2,56E-12	Origin recognition complex subunit 5	KOB72064.1
comp37345_c1_seq2	2,41	2,01E-24	4,43E-08	SYS1 homolog	NP_001040461.1
comp37465_c0_seq1	3,01	5,97E-14	5,37E-10	Zinc finger DNA binding	KOB73826.1
comp37465_c1_seq3	3,98	6,99E-27	2,63E-15	RNA-directed DNA polymerase from mobile element jockey-like	XP_013201040.1
comp37510_c0_seq1	-5,66	2,94E-70	1,82E-32	probable dolichol-phosphate mannosyltransferase	XP_013191674.1
comp37510_c0_seq2	6,27	8,81E-90	1,23E-39	probable dolichol-phosphate mannosyltransferase	XP_013191674.1
comp37573_c2_seq11	-4,71	2,53E-41	5,21E-26		
comp37573_c2_seq8	-2,84	4,96E-12	3,96E-12		
comp37723_c0_seq1	-6,13	2,07E-88	2,58E-36	vacuolar sorting-associated 28 homolog	XP_004933718.2
comp37723_c0_seq2	6,70	1,38E-111	3,87E-44	vacuolar sorting-associated 28 homolog	XP_004933718.2
comp37744_c0_seq1	-5,61	6,12E-64	3,99E-35		
comp37744_c0_seq2	4,34	4,11E-31	8,25E-25		
comp37935_c0_seq1	-2,51	1,91E-12	4,07E-05	RNA-directed DNA polymerase from mobile element jockey-like	XP_011559174.1
comp38088_c0_seq4	-5,64	9,20E-80	5,55E-33	lipase 3-like	AIN34700.1
comp38088_c0_seq5	-6,22	1,92E-105	2,43E-36	lipase 3-like	AIN34700.1
comp38091_c1_seq1	-2,10	1,28E-11	3,96E-04	KH domain- RNA- signal transduction-associated 2-like isoform X1	XP_013161826.1
comp38189_c0_seq1	2,37	3,59E-08	9,05E-07	myrosinase 1-like	XP_012545806.1
comp38330_c0_seq7	3,12	1,06E-14	5,09E-14	low-density lipo receptor-related 1 isoform X1	KPJ02992.1
comp38424_c3_seq7	-3,36	2,88E-18	1,62E-16	zinc finger BED domain-containing 6 isoform X1	JAT86786.1
comp38448_c0_seq4	-2,28	6,00E-09	6,63E-05	Activin receptor type-1	KPJ03040.1
comp38539_c0_seq4	6,34	1,50E-96	7,19E-39	KH domain- RNA- signal transduction-associated 2-like isoform X1	XP_013191603.1

comp38539_c0_seq5	-4,22	5,24E-32	1,62E-16	KH domain- RNA- signal transduction-associated 2-like isoform X1	XP_013191603.1
comp38579_c0_seq3	-2,31	6,30E-15	1,47E-04	pancreatic lipase-related 2-like	XP_004926042.1
comp38700_c0_seq2	-2,61	2,49E-61	3,64E-11	plasminogen receptor (KT)	JAT85718.1
comp38725_c0_seq1	2,17	9,38E-07	5,49E-07	trafficking particle complex subunit 12	XP_014359680.1
comp38921_c2_seq14	-3,59	7,89E-22	3,96E-12	TLD domain-containing 1	XP_014366726.1
comp38921_c2_seq9	2,63	5,54E-11	1,99E-06	TLD domain-containing 1	XP_013191625.1
comp38985_c0_seq7	-3,05	3,56E-14	5,44E-12	Uridine 5 -monophosphate synthase	XP_014365440.1
comp39047_c0_seq3	-2,16	7,98E-08	3,68E-06	glutathione synthetase-like isoform X1	XP_004927647.1
comp39047_c0_seq5	2,50	4,79E-27	2,16E-09	glutathione synthetase-like isoform X1	EHJ71631.1
comp39047_c0_seq7	4,26	4,45E-40	2,72E-14	glutathione synthetase-like	XP_013191677.1
comp39109_c1_seq17	3,16	8,08E-16	2,12E-12	zinc finger BED domain-containing 1-like	XP_011568411.1
comp39157_c0_seq8	2,31	4,28E-34	2,67E-09	mutant cadherin	ACY69027.1
comp39291_c0_seq1	2,30	9,13E-15	3,26E-06	phosphatidylinositide phosphatase SAC1	XP_013169730.1
comp39435_c1_seq17	2,13	1,64E-06	2,59E-06	esterase FE4-like	AFO65061.1
comp39453_c1_seq13	4,03	5,97E-28	4,33E-15	probable aminopeptidase NPEPL1	XP_013193880.1
comp39453_c1_seq8	-3,88	2,42E-26	6,66E-14	probable aminopeptidase NPEPL1	XP_013193880.1
comp39454_c1_seq6	-2,57	1,21E-09	1,73E-12	probable peptide chain release factor C12orf65 mitochondrial	KOB67490.1
comp39529_c4_seq11	-3,64	8,14E-22	8,58E-14	Ubiquitin-conjugating enzyme E2 T	JAT79627.1
comp39529_c4_seq13	3,39	1,46E-18	4,71E-11	Ubiquitin-conjugating enzyme E2 T	JAT79627.1
comp39535_c0_seq4	-2,57	7,54E-10	1,06E-07	piggyBac transposable element-derived 4-like	XP_013191790.1
comp39572_c0_seq6	-3,16	6,18E-20	8,12E-08	oxysterol-binding -related 6 isoform X1	XP_013193895.1
comp39743_c0_seq11	4,03	9,79E-28	2,99E-15	NF-kappa-B inhibitor-interacting Ras	XP_013137938.1
comp39743_c0_seq14	-4,18	5,53E-32	2,88E-15	NF-kappa-B inhibitor-interacting Ras	XP_013137938.1
comp39855_c0_seq2	2,43	4,58E-09	1,95E-08	reverse transcriptase	BAD86655.1
comp39873_c1_seq2	5,58	7,05E-57	2,30E-38	lipase member H-A-like	ANJ42864.1
comp39873_c1_seq3	-7,87	2,55E-170	5,39E-58	lipase member H-A-like	ANJ42864.1
comp39919_c2_seq4	-2,20	9,51E-25	4,34E-06	coiled-coil domain-containing 102A	XP_013137922.1

comp39919_c2_seq5	4,04	1,99E-29	1,40E-13	coiled-coil domain-containing 102A	KOB68643.1
comp39983_c1_seq2	2,91	4,38E-14	6,47E-10	Pacifastin-like protease inhibitor cvp4	JAT90547.1
comp39983_c1_seq5	-6,04	1,39E-78	2,27E-39	Pacifastin-like protease inhibitor cvp4	EHJ70585.1
comp40064_c1_seq3	3,59	5,97E-22	4,52E-11	nuclease HARBI1	XP_014359623.1
comp40086_c0_seq3	4,51	1,13E-73	5,55E-26	E3 ubiquitin- ligase MARCH5	XP_013181610.1
comp40086_c0_seq8	-4,04	1,27E-103	3,02E-23	E3 ubiquitin- ligase MARCH5	XP_013181610.1
comp40196_c2_seq2	3,33	1,01E-17	5,70E-11	Neuropilin and tolloid 2	KOB65391.1
comp40196_c2_seq9	-3,46	8,33E-26	2,72E-10	Neuropilin and tolloid 2	XP_013137871.1
comp40272_c0_seq3	-2,02	7,84E-10	2,62E-05	blastopia poly	XP_015435727.1
comp40291_c2_seq2	-2,48	7,84E-10	2,53E-05	histone-arginine methyltransferase CARMER isoform X2	XP_013200047.1
comp40323_c0_seq1	-2,26	5,69E-15	7,98E-08	Retrovirus-related Pol poly from transposon	EFA13518.1
comp40323_c0_seq5	-2,31	1,66E-23	4,07E-09	Retrovirus-related Pol poly from transposon	XP_012547467.1
comp40442_c0_seq9	-2,76	2,14E-46	8,52E-13	ran-binding 9	XP_012544190.1
comp40594_c0_seq1	-3,10	6,20E-15	7,38E-11	carbonyl reductase [NADPH] 1-like	XP_014367812.1

Supplementary Table S3: *p* values of the post-hoc tests of the difference in expression of candidate genes between *H. virescens,* VV, *H. subflexa* and SS

p values for Games-Howell or Tukey posthoc tests comparing expression (number of targets per 1000 reference) of candidate genes in pheromone glands of *H. subflexa* (Hsub), *H. virescens* (Hvir), VV or SS females.

	LipX	LipZ	Est1	Lip39873_A
Test performed	Games-Howell	Tukey	Tukey	Games-Howell
Hsub vs Hvir	0.266	**$2.58x10^{-09}$**	0.155	0.271
Hsub vs SS	0.402	1.000	0.989	0.344
Hsub vs VV	**0.010**	**$3.32x10^{-8}$**	**$1.34x10^{-3}$**	**$3.03x10^{-4}$**
Hvir vs SS	0.507	**$2.38\ x10^{-9}$**	0.254	0.588
Hvir vs VV	0.721	0.420	0.203	0.789
SS vs VV	**0.028**	**$3.03\ x10^{-8}$**	**$2.69\ x10^{-3}$**	**$1.96\ x10^{-3}$**

Supplementary Table S4: Mapping of differentially expressed contigs from the DD23 reference pheromone gland transcriptome to the chromosomes of *Bombyx mori*.

Name	Transcript ID	Bmor Hit ID	Chr	Bmor contig	Start	End	Blast2GO Description
	comp40594_c0_seq1	XP_004929128.1	4	NC_051361.1	66605	67558	carbonyl reductase [NADPH] 1-like
	comp40323_c0_seq1	XP_037867208.1	5	NC_051362.1	6990562	6996918	Retrovirus-related Pol poly from transposon
	comp40323_c0_seq5	XP_037867208.1	5	NC_051362.1	6990562	6996918	Retrovirus-related Pol poly from transposon
	comp39855_c0_seq2	XP_037867647.1	6	NC_051363.1	11608714	11612347	reverse transcriptase
	comp39109_c1_seq17	XP_037868454.1	8	NC_051365.1	7492339	7497438	zinc finger BED domain-containing 1-like
	comp40064_c1_seq3	XP_037868939.1	9	NC_051366.1	2251174	2253568	nuclease HARBI1
	comp39454_c1_seq6	XP_012549535.1	10	NC_051367.1	4004848	4007759	probable peptide chain release factor C12orf65 mitochondrial
	comp30551_c0_seq2	ADI61832.1	15	NC_051372.1	18288306	18289085	endonuclease-reverse transcriptase
	comp37465_c0_seq1	XP_037872948.1	17	NC_051374.1	1409032	1418748	Zinc finger DNA binding
	comp39157_c0_seq8	XP_037872904.1	17	NC_051374.1	10641965	10643209	mutant cadherin
	comp40442_c0_seq9	XP_012544189.2	19	NC_051377.1	10217296	10240717	ran-binding 9
	comp39535_c0_seq4	XP_012544199.1	19	NC_051376.1	13143201	13150502	piggyBac transposable element-derived 4-like
	comp36176_c0_seq3	XP_012551069.1	**20**	NC_051377.1	4772099	4775045	Diguanylate cyclase with PAS sensor
	comp35374_c0_seq1	XP_004922588.1	**20**	NC_051377.1	4833082	4836694	tetratricopeptide repeat 4
	comp35374_c0_seq2	XP_004922588.1	**20**	NC_051377.1	4833082	4836694	tetratricopeptide repeat 4
	comp39047_c0_seq3	XP_004927647.1	**20**	NC_051377.1	8601662	8631835	glutathione synthetase-like isoform X1
	comp39047_c0_seq5	XP_004927647.1	**20**	NC_051377.1	8601662	8631835	glutathione synthetase-like isoform X1
	comp39047_c0_seq7	XP_004927647.1	**20**	NC_051377.1	8601662	8631835	glutathione synthetase-like
	comp39983_c1_seq2	XP_037873936.1	**20**	NC_051377.1	8672224	8710983	Pacifastin-like protease inhibitor cvp4
	comp39983_c1_seq5	XP_037873938.1	**20**	NC_051377.1	8672224	8710983	Pacifastin-like protease inhibitor cvp4
	comp24491_c0_seq3	XP_021204406.2	**20**	NC_051377.1	8713974	8719052	Pacifastin-like protease inhibitor cvp4
	comp38700_c0_seq2	XP_004927282.1	**20**	NC_051377.1	8797679	8802120	plasminogen receptor (KT)
	comp37510_c0_seq1	XP_012546772.1	**20**	NC_051377.1	8851911	8856255	probable dolichol-phosphate mannosyltransferase

	comp37510_c0_seq2	XP_012546772.1	**20**	NC_051377.1	8851911	8856255	probable dolichol-phosphate mannosyltransferase
	comp39743_c0_seq11	XP_004927287.1	**20**	NC_051377.1	8856725	8862021	NF-kappa-B inhibitor-interacting Ras
	comp39743_c0_seq14	XP_004927287.1	**20**	NC_051377.1	8856725	8862021	NF-kappa-B inhibitor-interacting Ras
Est1	comp39435_c1_seq17	NP_001177297.1	**20**	NC_051377.1	8881451	8885519	esterase FE4-like
	comp36091_c0_seq1	XP_037874021.1	**20**	NC_051377.1	9094473	9110390	snRNA-activating complex subunit 1
	comp36091_c0_seq2	XP_037874021.1	**20**	NC_051377.1	9094473	9110390	snRNA-activating complex subunit 1
	comp39919_c2_seq4	XP_012545821.1	**20**	NC_051377.1	9113038	9120324	coiled-coil domain-containing 102A
	comp39919_c2_seq5	XP_012545821.1	**20**	NC_051377.1	9113038	9120324	coiled-coil domain-containing 102A
	comp36300_c0_seq1	XP_037873953.1	**20**	NC_051377.1	9157596	9227880	NACHT domain- and WD repeat-containing 1
	comp38921_c2_seq14	XP_037873980.1	**20**	NC_051377.1	9241699	9285874	TLD domain-containing 1
	comp38921_c2_seq9	XP_037873980.1	**20**	NC_051377.1	9241699	9285874	TLD domain-containing 1
	comp35971_c1_seq1	XP_037873836.1	**20**	NC_051377.1	9367668	9393926	f-box lrr-repeat 7
	comp35589_c0_seq1	XP_037873904.1	**20**	NC_051377.1	9397493	9413821	probable ATP-dependent RNA helicase DDX27
	comp35589_c0_seq2	XP_037873904.1	**20**	NC_051377.1	9397493	9413821	probable ATP-dependent RNA helicase DDX27
	comp39291_c0_seq1	XP_037873906.1	**20**	NC_051377.1	9414365	9433483	phosphatidylinositide phosphatase SAC1
	comp38539_c0_seq4	XP_037873903.1	**20**	NC_051377.1	9491084	9499550	KH domain- RNA- signal transduction-associated 2-like isoform X1
	comp38539_c0_seq5	XP_037873903.1	**20**	NC_051377.1	9491084	9499550	KH domain- RNA- signal transduction-associated 2-like isoform X1
	comp38091_c1_seq1	XP_012545817.1	**20**	NC_051377.1	9591571	9871758	KH domain- RNA- signal transduction-associated 2-like isoform X1
	comp31652_c0_seq3	XP_037874096.1	**20**	NC_051377.1	10217296	10240717	myrosinase 1-like
	comp28829_c0_seq1	XP_037874096.1	**20**	NC_051377.1	10217296	10240717	myrosinase 1-like
	comp38189_c0_seq1	XP_037874096.1	**20**	NC_051377.1	10217296	10240717	myrosinase 1-like
	comp40291_c2_seq2	XP_012545804.2	**20**	NC_051377.1	10259078	10264320	histone-arginine methyltransferase CARMER isoform X2
	comp38725_c0_seq1	XP_037873851.1	**20**	NC_051377.1	10275515	10287862	trafficking particle complex subunit 12
	comp37345_c1_seq2	NP_001040461.1	**20**	NC_051377.1	10319538	10323484	SYS1 homolog
Lip39873	comp39873_c1_seq2	XP_004926202.1	**20**	NC_051377.1	10367054	10407200 \	lipase member H-A-like
Lip39873	comp39873_c1_seq3	XP_004926202.1	**20**	NC_051377.1	10367054	10407200 \	lipase member H-A-like
	comp40196_c2_seq2	XP_037873850.1	**20**	NC_051377.1	10506954	10740684	Neuropilin and tolloid 2

	comp40196_c2_seq9	XP_037873850.1	**20**	NC_051377.1	10506954	10740684	Neuropilin and tolloid 2
	comp36915_c0_seq1	NP_001166865.1	**20**	NC_051377.1	10936402	10937912	Origin recognition complex subunit 5
	comp36915_c0_seq2	NP_001166865.1	**20**	NC_051377.1	10936402	10937912	Origin recognition complex subunit 5
	comp39453_c1_seq13	XP_037873855.1	**20**	NC_051377.1	10939420	10952945	probable aminopeptidase NPEPL1
	comp39453_c1_seq8	XP_037873855.1	**20**	NC_051377.1	10939420	10952945	probable aminopeptidase NPEPL1
	comp39572_c0_seq6	XP_037873860.1	**20**	NC_051377.1	10960968	11010936	oxysterol-binding -related 6 isoform X1
	comp38448_c0_seq4	XP_004926032.1	**20**	NC_051377.1	11352447	11365081	Activin receptor type-1
	comp38985_c0_seq7	XP_004926034.1	**20**	NC_051377.1	11428819	11439399	Uridine 5 -monophosphate synthase
	comp39529_c4_seq11	XP_004926038.1	**20**	NC_051377.1	11597036	11598585	Ubiquitin-conjugating enzyme E2 T
	comp39529_c4_seq13	XP_004926038.1	**20**	NC_051377.1	11597036	11598585	Ubiquitin-conjugating enzyme E2 T
	comp34863_c0_seq1	XP_004926039.1	**20**	NC_051377.1	11599280	11602112	kxDL motif-containing CG10681
	comp34863_c0_seq2	XP_004926039.1	**20**	NC_051377.1	11599280	11602112	kxDL motif-containing CG10681
	comp27469_c0_seq2	XP_037874016.1	**20**	NC_051377.1	11735463	11740431	hypothetical protein KGM_06068
Lip38579	comp38579_c0_seq3	XP_004926042.1	**20**	NC_051377.1	11789177	11810694	pancreatic lipase-related 2-like
	comp37723_c0_seq1	XP_037873998.1	**20**	NC_051377.1	11897816	11902117	vacuolar sorting-associated 28 homolog
	comp37723_c0_seq2	XP_037873998.1	**20**	NC_051377.1	11897816	11902117	vacuolar sorting-associated 28 homolog
	comp35597_c0_seq2	XP_037874119.1	**20**	NC_051377.1	12005591	12183381	GTP-binding RAD
LipX	comp38088_c0_seq4	XP_021204440.2	**20**	NC_051377.1	12093937	12095974	lipase 3-like
LipZ	comp38088_c0_seq5	XP_021204440.2	**20**	NC_051377.1	12093937	12095974	lipase 3-like
	comp36335_c1_seq1	XP_037873946.1	**20**	NC_051377.1	12224411	12234330	DNA ligase 1
	comp36335_c1_seq3	XP_037873946.1	**20**	NC_051377.1	12224411	12234330	DNA ligase 1
	comp38330_c0_seq7	XP_037873945.1	**20**	NC_051377.1	12234320	12304196	low-density lipo receptor-related 1 isoform X1
	comp32219_c0_seq1	XP_004927331.1	20	NC_051377.1	12323193	12325311	SOSS complex subunit B homolog
	comp37465_c1_seq3	XP_037874873.1	22	NC_051379.1	14565682	14568066	RNA-directed DNA polymerase from mobile element jockey-like
	comp37935_c0_seq1	XP_037874873.1	22	NC_051379.1	14565682	14568066	RNA-directed DNA polymerase from mobile element jockey-like
	comp36643_c0_seq11	XP_004924904.1	24	NC_051381.1	3653370	3662288	THAP domain-containing 1-like
	comp38424_c3_seq7	XP_012545005.1	24	NC_051381.1	17175771	17185051	zinc finger BED domain-containing 6 isoform X1

	comp40272_c0_seq3	XP_037876904.1	27	NC_051384.1	7671148	7673595	blastopia poly
	comp40086_c0_seq3	XP_037877359.1		NW_023637948.1	6966	20620	E3 ubiquitin- ligase MARCH5
	comp40086_c0_seq8	XP_037877359.1		NW_023637948.1	6966	20620	E3 ubiquitin- ligase MARCH5
	comp12227_c0_seq1	none					
	comp32945_c0_seq1	none					
	comp34660_c3_seq2	none					
	comp36702_c0_seq1	none					SDE2 homolog
	comp36702_c0_seq2	none					SDE2 homolog
	comp37573_c2_seq11	none					
	comp37573_c2_seq8	none					
	comp37744_c0_seq1	none					
	comp37744_c0_seq2	none					

Supplementary Table S5: Differential expression between SS and VV females of transcript sequences of the genes belonging the Chromosome 20 introgressed region of DD23

Results of differential expression analyse performed using DESeq2 and edgeR comparing SS and VV female pheromone glands for lipases and esterase genes in the introgressed region of Chr20. Metrics of the base 2 or natural logarithm of the fold change of expression and the associated adjusted *p* values are displayed as outputed by the tools (see Love *et al.* 2014 and Robinson *et al.* 2009).

	DESeq2		edgeR	
Gene ID	Log2FC	Adj. *p* value	logFC	FDR
Est1	**-2.49**	**1.01E-14**	**-2.73**	**3.77E-10**
Est2	0.37	5.99E-01	0.19	8.00E-01
Lip38579	-1.00	**2.81E-02**	-1.29	8.64E-02
Lip39873	-0.74	7.98E-02	-1.01	2.31E-01
LipA	**-3.04**	**2.44E-02**	**-3.20**	5.21E-02
LipB	0.63	2.57E-01	0.35	5.83E-01
LipX	-1.13	**5.12E-03**	-1.36	**1.28E-02**
LipY	**6.01**	**1.73E-05**	**6.23**	**3.89E-05**
LipZ	**-10.94**	**6.30E-30**	**-10.94**	**2.50E-61**

Supplementary Table S6: *p* values of the post-hoc tests of the difference in pheromone composition between the different CRISPR lines

p values for Games-Howell or Tukey posthoc tests comparing metrics of pheromone content comparing *H. subflexa* DD23 females that are bearing one wild-type chromosome 20 and one chromosome 20 that is either : wild-type (SS), beging a introgression of *H. virescens* (VS) or the same introgression for which candidate lipases (Lip s/-), esterases (Lip s/-) or both (Lip+Est s/-)have been knocked-out.

	Acetate amount	Z9-16:Ald amount	Z11-16:Ald amount	Z11-16:OH amount	14:Ald amount	Pheromone Amount
Test perfomed	Games-Howell	Games-Howell	Games-Howell	Games-Howell	Games-Howell	Games-Howell
Lip+Est s/- vs Est s/-	**0.017**	0.938	0.999	0.997	0.735	0.66
Lip s/- vs Est s/-	**0.009**	0.345	0.969	1	0.353	0.58
SS vs Est s/-	**1.16x10^{-05}**	0.17	1	0.934	**0.044**	0.19
VS vs Est s/-	0.545	0.395	**0.012**	**0.009**	0.996	**0.02**
Lip s/- vs Lip+Est s/-	0.82	0.68	0.95	1	0.666	0.99
SS vs Lip+Est s/-	0.607	0.681	1	0.78	0.322	0.97
VS vs Lip+Est s/-	**0.001**	0.596	**0.031**	**0.012**	0.966	0.16
SS vs Lip s/-	1	0.995	0.968	0.897	0.999	1
VS vs Lip s/-	**0.002**	0.986	**0.007**	**0.012**	0.501	0.49
VS vs SS	**4.93x10^{-07}**	0.92	**0.013**	**0.005**	0.196	0.32

	Relative % of acetates	Relative % of Z9-16:Ald	Relative % of Z11-16:Ald	Relative % of Z11-16:OH	Relative % of 14:Ald
Test perfomed	Games-Howell	Games-Howell	Tukey	Games-Howell	Tukey
Lip+Est s/- vs Est s/-	**0.003**	0.88	**0.007**	1	0.761
Lip s/- vs Est s/-	**1.6x10^{-04}**	0.923	**4.90x10^{-05}**	0.349	0.234
SS vs Est s/-	**1.44x10^{-07}**	0.953	**8.56x10^{-04}**	**0.001**	**0.036**
VS vs Est s/-	**1.2x10^{-04}**	0.943	0.807	0.219	0.170

Lip s/- vs Lip+Est s/-	0.913	0.598	0.482	0.623	0.863
SS vs Lip+Est s/-	0.802	0.599	0.988	**0.014**	0.521
VS vs Lip+Est s/-	**1.92x10^{-07}**	1	**0.003**	0.198	**0.022**
SS vs Lip s/-	1	0.999	0.737	0.104	0.994
VS vs Lip s/-	**8.39x10^{-08}**	0.726	**4.53x10^{-05}**	**0.04**	**0.003**
VS vs SS	**1.1x10^{-12}**	0.761	**6.48x10^{-04}**	**0.006**	**2.43x10^{-4}**

	Acetate amount log contrasted to 14:Ald	Z9-16:Ald log contrasted to 14:Ald	Z11-16:Ald log contrasted to 14:Ald	Z11-16:OH log contrasted to 14:Ald
Stat. test perfomed	Games-Howell	Tukey	Games-Howell	Games-Howell
Lip+Est s/- vs Est s/-	0.246	0.255	0.088	0.869
Lip s/- vs Est s/-	**0.043**	0.481	**2.74x10^{-04}**	0.136
SS vs Est s/-	**0.021**	0.256	**0.002**	**0.002**
VS vs Est s/-	0.418	0.407	0.136	0.052
Lip s/- vs Lip+Est s/-	0.991	1	0.132	0.266
SS vs Lip+Est s/-	0.984	1	0.443	**0.004**
VS vs Lip+Est s/-	**0.012**	**0.013**	**7.43x10^{-04}**	0.006
SS vs Lip s/-	1	1	0.975	0.432
VS vs Lip s/-	**0.003**	**0.038**	**1.92x10^{-05}**	**5.39x10^{-04}**
VS vs SS	**0.002**	**0.048**	**4.64x10^{-05}**	**4.21x10^{-05}**

Supplementary Table S7: Primers used for sequencing candidate genes

Table display the sequences of the primers used to confirm the ORF sequence of candidate genes and for CRISPR/Cas9 related experiments.

ORF sequencing primers	
Name	Sequence
LipX-SS-F1	CTTCACCGAACTCACCGCT
LipX-SS-F2	CCAACACGTTTAAGGTCCTGC
LipX-SS-R1	CCACTATTCATACTCTGTCCGTAC
LipX-SS-R2	CAGTCTCTCAGTCACGCCTG
LipX-VV-F1	GACATGGCTCCGATACAATCA
LipX-VV-F2	TCCTGTTCTCAACCTTCGCA
LipX-VV-R1	GTTCATACTCTGTCCATAACGTG
LipX-VV-R2	CAGTCTCTCAGTCACGCCTG
LipZ-VV-F1	CAGCCACTCCACGTCTCTTA
LipZ-VV-F3	GGTTCTGCTCCTTCCCGTAG
LipZ-VV-R2	GCTAAGTCTTCGCTCGGCTA
LipZ-VV-R4	AGTTGATGACATGGCTCCGA
38579_0_1-SS-F1	ACCCGTAGCTCATCCAAGTG
38579_0_1-SS-R2	GTGTTGATATCGCTGGTCTGC
38579_0_3-VV-F1	GGTCCTCCCAGTGCATTTTG
38579_0_3-VV-R2	GCTGGTGTTGATATCGCTGG
39435_1_A-SS-F1	CGGATAATAAAACTGTGATTGTGG
39435_1_A-SS-F2	CTATGGCGGAGGAGGAGTA
39435_1_A-SS-R1	CGTCACATTCTGGTAGTCACTC
39435_1_A-SS-R2	ATTCACCGACCATTCAGCTC
39435_1_A-VV-F1	TGAAGATACGGTTACTGTGCAG
39435_1_A-VV-F2	GCAATAGTTCAGAGTGGCTCC
39435_1_A-VV-R1	CACTTCCTCCTATGTACGAGAAC
39435_1_A-VV-R2	CGCTTTCTGTGCCATGTATC
SS-39873_1-ORF-F1	CAGGCCGATCAAAATGTGCT
SS-39873_1-ORF-R2	ACACTGGTGAGTCAAGCCC
SS-39873_1-ORF-R4	GATCTGCTGACACTAGCGCT
VV-39873_1-ORF-F1	ACACTGGGCAAATCTAGCCT
VV-39873_1-ORF-R2	GATCTGCTGACACTAGCGCT
qPCR primers	
Name	Sequence
LipX-F1	GTCTCTCAGTCACGCCTG
LipX-R2	TGGAAAGCGTGTTATCAATGACA
LipZ-F1	CACCAGAAATGTCCCAGTGC
LipZ-R2	CCCGCGATGATAGACCATGT
38579_0_A-F1	GCTCCACGCCAATGAAGC
38579_0_A-R2	CTCCAAGGGCAGCTTCTCC
39435_1_A-F1	GCGTCAGTATCATGCTCAAGT
39435_1_A-R2	GCTGCTATCAAACTTGCCAAAT
39873_1_A-F1	GTTGAAATTCCCCTCCATTAGCA

39873_1_A-R2	GGACCTTGCTTCCGAAGTCT
Screening Primers (CRISPR)	
Name	Sequence
VVest1-scr-Fd	CCTGCAGTGTCTCMGACTCAA
VVest1-scr-R	GCCATAGCTTTCACCAGCA
VVest2-scr-F	CCACATACAGCCAACGAGAA
VVest2-scr-R	ATGCAAATCTACGCCACCTC
VVLipX-scr-Fd	CGATGATCGACYATGTTCTGGA
VVLipX-scr-R	AAGCCGCCTTTAGGAAACAC
VVLipZ-scr-F	GATGGGAGTCTTCGATGTGC
VVLipZ-scr-R	GGCAGAAGAATGCACCAAA
Guide RNA (CRISPR)	
Name	Sequence
VVLipX-T1	CCAAGGAACGGGAACATTCC
VVLipX-T2	AGTCATAGTCCTAAATATCC
VVLipZ-T1	TCAAGGCACTGGGACATTTC
VVLipZ-T2	GAATTTTTATGTCTACTTGC
VVest1-T1	TGTCTTGTTTGACCAAATAT
VVest1-T2	CGGAATGTCACTGGCAATCA
VVest2-T1	TGTCGTGTTTGACCAAATGT
VVest2-T2	ATGGATAAGGAAAAATATAG
