# Supplementary material for: Lipases and carboxylesterases affect moth sex pheromone compounds involved in interspecific mate recognition
Source: Nat Commun. 2023 Nov 18;14:7505. doi: 10.1038/s41467-023-43100-w (PMC10657362; doi:10.1038/s41467-023-43100-w)
Supplement: Supplementary file 2 — Reporting Summary [file 41467_2023_43100_MOESM2_ESM.pdf]

nature portfolio

Corresponding author(s): Arthur DE FOUCHIER

Last updated by author(s): Oct 17, 2023

Reporting Summary

Nature Portfolio wishes to improve the reproducibility of the work that we publish. This form provides structure for consistency and transparency in reporting. For further information on Nature Portfolio policies, see our Editorial Policies and the Editorial Policy Checklist.

Statistics

For all statistical analyses, confirm that the following items are present in the figure legend, table legend, main text, or Methods section.

n/a	Confirmed	
☐	☒	The exact sample size (*n*) for each experimental group/condition, given as a discrete number and unit of measurement
☐	☒	A statement on whether measurements were taken from distinct samples or whether the same sample was measured repeatedly
☐	☒	The statistical test(s) used AND whether they are one- or two-sided<br>*Only common tests should be described solely by name; describe more complex techniques in the Methods section.*
☐	☒	A description of all covariates tested
☐	☒	A description of any assumptions or corrections, such as tests of normality and adjustment for multiple comparisons
☐	☒	A full description of the statistical parameters including central tendency (e.g. means) or other basic estimates (e.g. regression coefficient) AND variation (e.g. standard deviation) or associated estimates of uncertainty (e.g. confidence intervals)
☐	☒	For null hypothesis testing, the test statistic (e.g. *F*, *t*, *r*) with confidence intervals, effect sizes, degrees of freedom and *P* value noted<br>*Give P values as exact values whenever suitable.*
☒	☐	For Bayesian analysis, information on the choice of priors and Markov chain Monte Carlo settings
☒	☐	For hierarchical and complex designs, identification of the appropriate level for tests and full reporting of outcomes
☒	☐	Estimates of effect sizes (e.g. Cohen's *d*, Pearson's *r*), indicating how they were calculated

Our web collection on statistics for biologists contains articles on many of the points above.

Software and code

Policy information about availability of computer code

Data collection	For qPCR : Applied Biosystem 7500 Real-Time PCR System software<br>For GC : Agilent ChemStation (version B.04.03)
Data analysis	R Studio version 1.0.136<br>R version 3.3.2<br><br>RNAseq assembly and analysis performed on the BIPAA Galaxy server using :<br>Trimmomatic, Ribopicker, Trinity, Bowtie2, RSEM, BUSCO, Transrate, Deseq2, edgeR & Blast2GO<br><br>Docking analysis :<br>ColabFold<br>PrediSi<br>AutoDockTools from MGLTools v1.5.7<br>Vina v1.2.3<br>Chimera X 1.4

For manuscripts utilizing custom algorithms or software that are central to the research but not yet described in published literature, software must be made available to editors and reviewers. We strongly encourage code deposition in a community repository (e.g. GitHub). See the Nature Portfolio guidelines for submitting code & software for further information.

Data

Policy information about availability of data

All manuscripts must include a data availability statement. This statement should provide the following information, where applicable:

- Accession codes, unique identifiers, or web links for publicly available datasets
- A description of any restrictions on data availability
- For clinical datasets or third party data, please ensure that the statement adheres to our policy

RNAseq reads have been deposited on NCBI Sequence Read Archives, accession numbers ERX682290 to ERX682297. Sequences resulting from genomic DNA Sanger sequencing are available on Genbank, accession numbers OK556469 to OK556476. Raw pheromone and qPCR data have been deposited on figshare (doi attribution pending).

Research involving human participants, their data, or biological material

Policy information about studies with human participants or human data. See also policy information about sex, gender (identity/presentation), and sexual orientation and race, ethnicity and racism.

Reporting on sex and gender	Non applicable
Reporting on race, ethnicity, or other socially relevant groupings	Non applicable
Population characteristics	Non applicable
Recruitment	Non applicable
Ethics oversight	Non applicable

Note that full information on the approval of the study protocol must also be provided in the manuscript.

Field-specific reporting

Please select the one below that is the best fit for your research. If you are not sure, read the appropriate sections before making your selection.

☒ Life sciences ☐ Behavioural & social sciences ☐ Ecological, evolutionary & environmental sciences

For a reference copy of the document with all sections, see nature.com/documents/nr-reporting-summary-flat.pdf

Life sciences study design

All studies must disclose on these points even when the disclosure is negative.

Sample size	Sample size were determined by following concensial recommended pratices in our field and balanced with eventual difficulties in sampling enough animals we used moths lines for which some genotypes are difficult to acquire in high number over a short period of time. Our sample size appears sufficient as the variability of most groups is low.
Data exclusions	One data point was excluded for qPCR of Hvir Est1 as the sample gave Ct value equivalent to those of the reference gene, suggesting a technical problem.<br>Samples for which the genotyping PCR didn't worked were excluded (12 samples). Pheromone samples that had a low amount of pheromone were excluded as well the results of their integration might be inaccurate. We used 50ng as treshold for the pheromone data collected on DD23 individuals, as only 4 samples were removed by this action and 40ng for the CRISPR pheromone data which removed 5 samples (instead of 12 if we would have used a treshold of 50). One VV_DD23 sample was removed because incoherent pheromone composition (the major component only represented 12% of the pheromone blend) which might have been caused by an issue during the pheromone gland extraction or while the sample was processed in the GC).
Replication	Difference in levels of acetates in D023 female pheromone between the genotypes have been assessed multiple time during the generation and the rearing of this line of Heliothis subflexa over multiple year, which always gave coherent results. Differential expression analyses of DD23 female pheromone glands and associated qPCR experiments have not been replicated, as they are time and ressource consuming and confirm each others. Pheromone extraction on lipases and esterase KO animals were not replicated due to the difficulties of obtaining indepently similar mutant lines. Docking experiment have not been replicated as they are automated in sillico analyses have been designed to limit randomness.
Randomization	Organisms were put in experimental groups based on their genotypes, obtained through genotyping of the animals prior to experiments.
Blinding	Experimentators were blind to the group of their samples when analysing chemical samples in GC. Not applicable for transcriptomic, qPCR, phylogenetic and docking analyses as thoose analyses can't be randomised and/or are automatically analysed by a software.

Reporting for specific materials, systems and methods

We require information from authors about some types of materials, experimental systems and methods used in many studies. Here, indicate whether each material, system or method listed is relevant to your study. If you are not sure if a list item applies to your research, read the appropriate section before selecting a response.

Materials & experimental systems

n/a	Involved in the study
☒	☐ Antibodies
☒	☐ Eukaryotic cell lines
☒	☐ Palaeontology and archaeology
☐	☒ Animals and other organisms
☒	☐ Clinical data
☒	☐ Dual use research of concern
☒	☐ Plants

Methods

n/a	Involved in the study
☒	☐ ChIP-seq
☒	☐ Flow cytometry
☒	☐ MRI-based neuroimaging

Animals and other research organisms

Policy information about studies involving animals; ARRIVE guidelines recommended for reporting animal research, and Sex and Gender in Research

Laboratory animals	Animals used in this study are all Lepidoptera: Heliothis subflexa females, wild type, DD23 strain and DD23 lipases and esterase KO strains. Heliothis virescens females. All animals were 2 to 4 days old. These strains are reared in the lab. No animals were captured in the wild recently. Animal where sacrified after experimentation by putting them at -20°C.
Wild animals	No wild animals were used in this study.
Reporting on sex	We report obserations on genes that are present in both male and female genome and on their expression in female sex pheromone glands as well as on female sex pheromone content..
Field-collected samples	No field samples were used in this study.
Ethics oversight	No ethical approval or guidance were required for this study as it used only insects no subject to ethical regulations.

Note that full information on the approval of the study protocol must also be provided in the manuscript.
