# Supplementary material for: Lipases and carboxylesterases affect moth sex pheromone compounds involved in interspecific mate recognition
Source: Nat Commun. 2023 Nov 18;14:7505. doi: 10.1038/s41467-023-43100-w (PMC10657362; doi:10.1038/s41467-023-43100-w)
Supplement: Supplementary file 3 — Description of Additional Supplementary Files [file 41467_2023_43100_MOESM3_ESM.pdf]

Description of Additional Supplementary Files

File Name: Supplementary Data 1
Description: Fasta file of the mapping targets for the differential expression analysis of introgressed genes.
